# PLATOX: Integrated In Vitro/In Vivo Approach for Screening of Adverse Lung Effects of Graphene-Related 2D Nanomaterials

**DOI:** 10.3390/nano12081254

**Published:** 2022-04-07

**Authors:** Otto Creutzenberg, Helena Oliveira, Lucian Farcal, Dirk Schaudien, Ana Mendes, Ana Catarina Menezes, Tatjana Tischler, Sabina Burla, Christina Ziemann

**Affiliations:** 1 Fraunhofer Institute for Toxicology and Experimental Medicine ITEM, 30625 Hannover, Germany; dirk.schaudien@item.fraunhofer.de (D.S.); tatjana.gripp@gmx.de (T.T.); 2 Department of Biology & CESAM, University of Aveiro, 3810-193 Aveiro, Portugal; holiveira@ua.pt (H.O.); anamendes@live.com.pt (A.M.); catarinamenezes@msn.com (A.C.M.); 3 BIOTOX SRL, 407280 Cluj-Napoca, Romania; lucian.farcal@biotox.ro (L.F.); sabina.burla@list.lu (S.B.); 4 Department of Environmental Research and Innovation, Luxembourg Institute of Science and Technology, 4422 Belvaux, Luxembourg

**Keywords:** graphene, 2D, nanoplates, lung, inhalation, toxicity, genotoxicity, in vitro, inflammation, hazard assessment

## Abstract

Graphene-related two-dimensional nanomaterials possess very technically promising characteristics, but gaps exist regarding their potential adverse health effects. Based on their nano-thickness and lateral micron dimensions, nanoplates exhibit particular aerodynamic properties, including respirability. To develop a lung-focused, in vitro/in vivo screening approach for toxicological hazard assessment, various graphene-related nanoplates, i.e., single-layer graphene (SLG), graphene nanoplatelets (GNP), carboxyl graphene, graphene oxide, graphite oxide and Printex 90^®^ (particle reference) were used. Material characterization preceded in vitro (geno)toxicity screening (membrane integrity, metabolic activity, proliferation, DNA damage) with primary rat alveolar macrophages (AM), MRC-5 lung fibroblasts, NR8383 and RAW 264.7 cells. Submerse cell exposure and material-adapted methods indicated material-, cell type-, concentration-, and time-specific effects. SLG and GNP were finally chosen as in vitro biologically active or more inert graphene showed eosinophils in lavage fluid for SLG but not GNP. The subsequent 28-day inhalation study (OECD 412) confirmed a toxic, genotoxic and pro-inflammatory potential for SLG at 3.2 mg/m^3^ with an in vivo-ranking of lung toxicity: SLG > GNP > Printex 90^®^. The in vivo ranking finally pointed to AM (lactate dehydrogenase release, DNA damage) as the most predictive in vitro model for the (geno)toxicity screening of graphene nanoplates.

## 1. Introduction

Graphene-related two-dimensional (2D) nanomaterials (nanoplates; GRNP) belong to the group of graphene-based engineered nanomaterials that are currently subject to an accelerated toxicological characterization. Before its first isolation from graphite by Novoselov and Geim in 2004, graphene was only a scientific model and known as a building block of already well-known carbon-based three-dimensional (3D) nanomaterials, such as graphite, carbon nanotubes (CNT) and fullerenes [1]. Graphene emerged as a 2D allotrope of carbon nanomaterials characterized by a single flat atom layer as a monocrystalline structure. Graphene is believed to be composed of benzene rings stripped of their hydrogen atoms. The carbon atoms are sp2-hybridized and hexagonally arranged, similar to a lattice honeycomb structure, with a C-C bond length of 0.142 nm [2,3]. Graphene belongs, as a pristine, defect-free, single-atom layer carbon plate, to the graphene-based family of nanomaterials (GFN), which also comprises graphene oxide (GO) as the most popular member, reduced graphene oxide (rGO), exfoliated graphene flakes, and graphene nanoplatelets (GNP) as well as the respective chemically functionalized versions [4]. Graphene nanoplates include single-layer (SLG), bilayer, trilayer and few-layer graphene (FLG; 3–10 well-defined stacked graphene layers) as well as graphene nanoplatelets (GNP) with more graphene layers [5,6].

GRNP are unique compared to spherical nanoparticles or one-dimensional (1D) carbon nanomaterials such as CNT or nanorods, owing to their physicochemical (PC) properties. GRNP possess unique features, e.g., high surface area per unit mass, excellent thermal and electric conductivity, and outstanding mechanical properties and strength. Their very promising PC properties have led to manifold applications in electronics, photonics, composite materials, catalysts, energy generation/storage, sensors, computer memory, metrology, and biomedicine [7,8]. However, the unique PC properties of GRNP can also influence interaction with cells and cellular substructures and, thus, their biocompatibility, with the probability to pose human health risks [9].

Given the large-scale use of GRNP in different industries (currently 1 to 10 tons per annum [10]), occupational exposure, but also exposure to consumers and the general population might occur. Appropriate risk assessment is, therefore, of high importance, in order to draw qualified conclusions upon the delicate balance between technology advancements and hazards to human health and the environment. However, according to the notifications provided by companies to ECHA during REACH registrations, no hazards have been classified for graphene [11]. Since large-scale production and commercialization of GRNP are imminent, the study of real hazards is indispensable. Even if the health effects associated with potential adversity of graphene have already been studied at the cellular level (in vitro) and in animal models (in vivo), as shown by the increasing number of research papers published on graphene toxicity and genotoxicity [2,5,12,13,14], the risk posed to humans remains nearly unexplored.

Despite the popular image of GRNP, resulting from their PC properties and the promising applications in various fields, many forms can be hazardous to human health, as they are processed as dry powders, thus posing a significant exposure risk at workplaces, in particular, through the inhalation route [15]. Humans can be exposed to graphene from various sources, mostly during manufacturing, with the potential risk of inhalation toxicity [16]. Therefore, the measurement of airborne dust levels in research laboratories and pilot and full-scale manufacturing facilities should be performed and integrated with aerodynamic diameters and deposition profiles from published theories on thin-plate aerodynamics [9]. Nanomaterials up to 25 µm in diameter can deposit ahead of the ciliated airways upon inhalation [17]. It is very difficult to figure out appropriate and real-life exposure scenarios leading to significant toxicity, as the PC properties of graphene are novel and have not been explored in depth in inhalation studies. However, a recent publication provides important data on human exposure during GNP production for research and development purposes, including real-time measurements and personal sampling. The publication finally identified the most critical production phases [18].

A review on inhalation testing of graphene for hazard assessment demonstrated that the existing database, especially for the inhalation route, is still too scarce to allow proper risk assessment [19]. Although important toxicological analogies may be available from other carbonaceous nanomaterials such as carbon black (CB) and CNT, direct extrapolation from those material classes to GRNP is not appropriate, as new engineered nanomaterials may pose unexpected risks [20]. In a 4-week inhalation test with rats at aerosol concentrations up to 1.9 mg of GNP per m^3^, no signs of inflammation were noticed at any time point [21]. Due to their nano-thickness, GNP and, more generally, GRNP exhibit particular aerodynamic properties, allowing deposition in the lung alveolar region. GRNP are nano-scaled in one dimension, whereas the other two dimensions are often micron-scaled. The specific aerodynamic consequences have been discussed in detail by [9]. Calculation of the deposition of GNP with 0.5, 5 and 25 µm lateral dimensions resulted in unexpectedly high deposition efficiencies of 45–10%. Compared to multiwalled carbon nanotubes (MWCNT, tangled type), GNP showed a lower toxic potential in a 5-day inhalation test, whereas no relevant toxicity was detected for CB and graphite nanoplates. Rigid MWCNT, however, showed a stronger toxic response than GNP [22]. After intravenous injection into mice, no hematotoxicity and no pro-inflammatory potential of highly water-dispersed FLG in lymph node and spleen cells were observed, thus pointing to high biocompatibility [13].

Due to the limited availability of in vivo data for pristine graphene nanoplates in particular and predictive toxicological in vitro screening approaches for GRNP in general, the development of integrated in vitro/in vivo screening approaches and filling of data gaps is inevitable to establish safe exposure levels. In-depth knowledge about graphene-based materials in terms of biocompatibility as well as comprehensive toxicological evaluation and risk analysis is imperative to ensure the safe application of these versatile materials. Risk analysis of graphene should be done by completing the following steps: in vitro and in vivo hazard assessment, risk assessment, risk characterization, risk communication, risk management, and policy relating to risk in the context of risks of concern to individuals, to the public- and private-sector organizations, and to society at a local, regional, national, or global level [23]. The main objective for human health risk assessments is to finally establish acceptable exposure levels for humans to specific GRNP.

In the EU-funded project PLATOX, eight mostly commercially available GRNP species were, therefore, selected, i.e., two SLG, two GNP, carboxyl graphene (CG), graphene oxide (GO) and graphite oxide, supplemented by technical soot (Printex 90^®^; non-platelet reference), to develop an integrated, lung-focused approach for proper hazard assessment of GRNP and to fill toxicological data gaps. Based on the 3R principles (Replacement, Reduction and Refinement) regarding animal experiments, it is also highly desirable to define in vivo-validated, predictive in vitro screening approaches for GRNP. As such, we screened different lung-relevant cell types, i.e., primary rat alveolar macrophages (AM), human MRC-5 lung fibroblasts, murine RAW 264.7 and rat NR8383 macrophages, using different endpoints/screening methods regarding their ability to predict in vivo lung toxicity of GRNP and, particularly, of pristine graphene nanoplates. In vitro results were finally validated by a 28-day (with 4 weeks of recovery) inhalation study with the pristine graphene nanoplate species that was least biologically active in vitro (GNP) and one of the two that were highly active in vitro (SLG).

When considering the in vivo results (with a clear cytotoxic, genotoxic, and pro-inflammatory potential of SLG, but less activity of GNP after 28 days of inhalation) AM as the primary rat cell type directly derived from the respiratory tract showed the highest reliability in predicting in vivo adverse lung effects of the pristine graphene nanoplates tested in the present study, with lactate dehydrogenase (LDH) release and induction of DNA strand breaks as most meaningful endpoints. It can be concluded from the study results that predictive in vitro lung-focused toxicity screening of GRNP samples seems, in principle, to be possible when adequately choosing relevant cell models and endpoints, appropriate dispersion methods, incubation times and concentrations, and carefully adapting photometric and immunological detection methods in particular to the specific GRNP materials.

## 2. Materials and Methods

### 2.1. Graphene-Related 2D Nanomaterials (GRNP)

#### 2.1.1. GRNP Species

For reasons of relevance, commercially available GRNP samples were chosen for the present study. Six GRNP samples, including three functionalized materials, were purchased from ACS Material, LLC (Medford, MA, USA). These samples comprised two single-layer graphene samples (SLG), graphene nanoplatelets (GNP), carboxyl graphene (CG), graphene oxide (GO) powder and graphite oxide. Additionally, an experimental GNP sample was provided by Avanzare (Logroño, Spain). Spherical carbon black (CB; Printex 90^®^; Evonik Industries AG, Essen, Germany), as a non-platelet reference, completed a total set of eight test samples, subsequently encoded P1-P8 (see Table 1).

#### 2.1.2. Material Characterization

**Figure 1:**
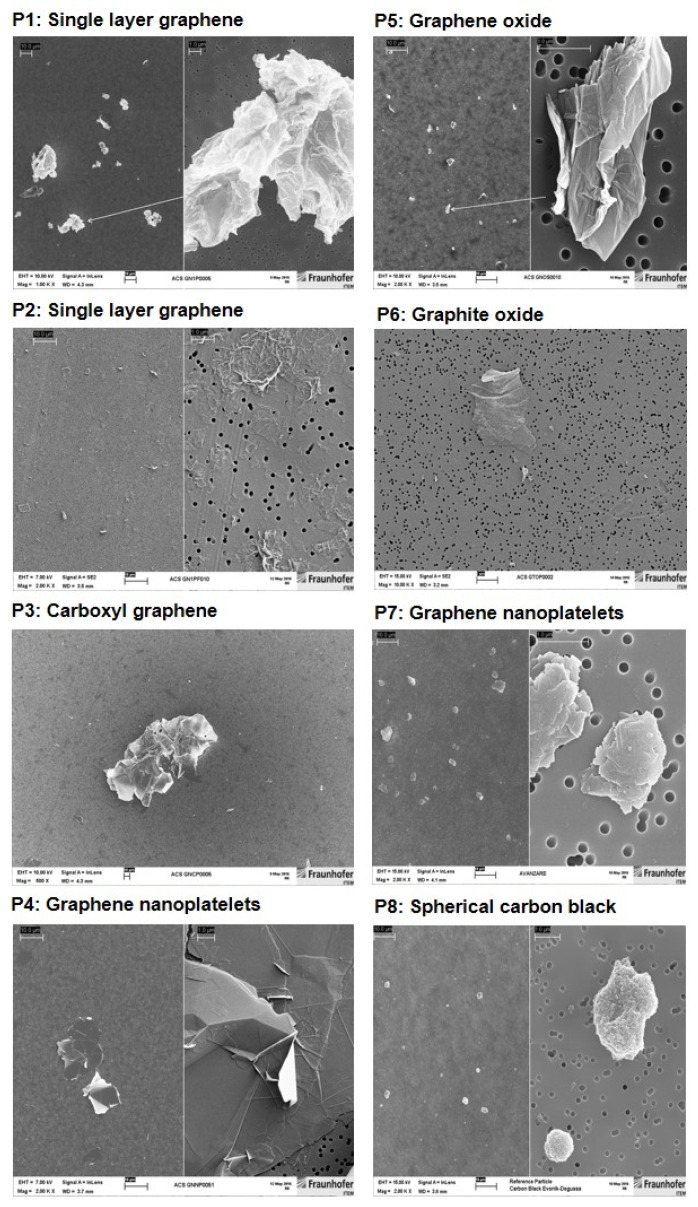
Scanning electron microscopic (SEM) pictures of the GRNP materials P1–P8, dispersed by sonication in cell culture medium with 10% FCS. Magnifications used for the split pictures were as follows, left: 1000–2000×; right: 20,000–50,000×. For the P3, a magnification of 500× was used, and for P6, a magnification of 10,000× was used.

To supplement the PC information provided by the suppliers of the materials, the specific surface area was re-evaluated as a service by the Fraunhofer Institute for Ceramic Technologies and Systems IKTS (Dresden, Germany), using the Brunauer–Emmett–Teller (BET) method. Part of the resulting data did not match the BET values given by the material suppliers (Table 1). Sterility testing of GRNP was done by adding a defined amount of the GRNP dispersions to thioglycolate broth (supporting the growth of a broad panel of bacteria and fungi) and incubating duplicate samples at 34–35 °C for 14 days. Physiological saline (0.9% NaCl) served as negative and Staphylococcus aureus (ATCC 25923) as a positive control. Turbidity was finally determined by the naked eye. Additionally, endotoxin content was measured by a commercial service laboratory (Lonza Bioscience, Verviers, Belgium) to avoid unspecific pro-inflammatory effects due to bacterial contamination. Both sterility and endotoxin testing did not point to relevant contaminations. Additionally, scanning electron microscopic (SEM; Zeiss Supra 55, Carl Zeiss NTS GmbH, Oberkochen, Germany) pictures (Figure 1) were generated for all samples to look for material morphology and dispersion grade. Therefore, stock dispersions of 50 µg/mL were prepared by ultrasonication in cell culture medium (in vitro experiments, see Section 2.1.3) or in a dispersion medium (in vivo study), as described by [24]. Depending on the material, magnifications in the range of 1000–50,000× were used.

#### 2.1.3. Dispersion and In Vitro Treatment with GRNP Materials

For in vitro (geno)toxicity screening and respective SEM analyses, the various GRNP were accurately weighed into glass vials and stock dispersions were prepared by suspending them in the cell type-specific cell culture media with 10% fetal calf serum (FCS), stirring the dispersions for 10 min using a magnetic stirrer and finally applying ultrasonication. At Fraunhofer ITEM, ultrasonication was performed by placing the dispersion on ice and applying sonication using a Sonoplus HD2070 (70 W) ultrasonic homogenizer and a VS70T sonotrode (diameter: 13 nm), both from Bandelin (Berlin, Germany). Ultrasonication was applied three times for 5 min each with 1 min breaks in between (90% duty cycle amplitude of 80 μm/ss and 0.9 s working intervals, 0.1 s rest intervals). A similar procedure was followed at the University of Aveiro, except a Vibra-Cell ultrasonic processor (Sonics & Materials, Inc., Newtown, CT, USA) was used. Appropriate dispersion was estimated by light microscopy. Subsequent pH measurements of the GRNP dispersions indicated slightly basic conditions for all samples, ranging from pH 8.18 (P5) to pH 8.50 (P7). For cell exposure, the stock dispersions were finally diluted to the double-concentrated (AM and NR8383) or final (other cell types) mass-based concentrations with the respective FCS-containing cell culture media, and dispersions were vortexed for 5 sec directly before cell treatment. Submerse cell treatment with the GRNP materials was done for 4–48 h, depending on the cell type and endpoint used.

#### 2.1.4. Sedimentation Kinetics

Sedimentation kinetics of the various GRNP species was estimated by determining the decay of turbidity using spectrophotometric OD600 measurements. Dispersions of the different GRNP (6.25 µg/mL, corresponding to the lowest concentration used for cell exposure, i.e., 3.125 µg/cm^2^) were prepared, as described under Section 2.1.3. One milliliter of FCS-containing cell culture medium (blank) and the GRNP dispersions were placed into disposable semi-micro spectrophotometric cuvettes and turbidity (OD600) was measured directly after material dispersion (time point zero) and after 1, 18, 24 and 48 h with an Eppendorf BioPhotometer^®^ (Eppendorf, Hamburg, Germany). In between measurements, cuvettes were stored vertically under cell culture conditions (37 °C, 5% CO_2_, in a water-saturated atmosphere) and were handled very carefully during measurements to avoid unspecific swirling up of the sedimented GRNP fractions. The time point zero OD600 values were set to 100% and relative OD600 values were calculated to enable a direct comparison of relative decays in turbidity for all GRNP materials.

### 2.2. In Vitro Screening

#### 2.2.1. Cell Models

For in vitro (geno)toxicity screening of the various GRNP and, in particular, the pristine GRNP, both lung-relevant primary/normal alveolar macrophages and lung fibroblasts and macrophage cell lines were used and evaluated for their ability to finally predict in vivo lung response.

Primary rat alveolar macrophages (AM) were chosen as the first primary lung-relevant cell model, as AM represent the first site of contact for particles in the lung and had previously been shown to represent a sensitive test system for the detection of membrane and direct DNA damage (DNA strand breaks, oxidative base modifications) as well as cytokine/chemokine release, when testing various particulate matter [25,26,27]. Cells were obtained from healthy female (nulliparous and non-pregnant) Wistar rats [strain Crl:WI (Han); Charles River Deutschland Co., Sulzfeld, Germany] by bronchoalveolar lavage (BAL) in compliance with the German Federal Act on the Protection of Animals [28], cultured and exposed to GRNP, as described previously [29]. After preparation, lungs were lavaged 3 times, each with 4 mL of 0.9% NaCl (brought to room temperature to avoid macrophage activation). Gentle massage enabled the isolation of sufficient cell numbers. After 10 min of centrifugation (300× *g*, 4 °C) of the lavages, cells were counted and plated at a density of 1.5 × 10^5^ cells per well on 24-well plates with hydrophobic culture surface (1.9 cm^2^ cell culture surface per well; Nunc, Thermo Fisher Scientific, Brunswick, Germany). Before being exposed to GRNP, cells were pre-cultured for 24 h in 500 µL of Dulbecco’s Modified Eagle Medium (DMEM) with high glucose (4.5 g/L), GlutaMax^TM^ and sodium pyruvate (110 mg/L), supplied by GIBCO, Thermo Fisher Scientific (Brunswick, Germany), and supplemented with 100 µg/mL streptomycin sulfate, 100 U penicillin G, sodium salt (Sigma-Aldrich, Taufkirchen, Germany) and 10% (*v*/*v*) FCS (Sigma-Aldrich, Taufkirchen, Germany) at 37 °C and 5% CO_2_ in a humidified atmosphere using an incubator. The GRNP materials were added to the cultures two-fold concentrated in a volume of 500 µL without medium exchange to avoid unspecific cell activation. Cells were incubated for 4 or 24 h with the test materials prior to endpoint analysis.

As the second lung-relevant cell type, human MRC-5 fetal lung fibroblasts were used for in vitro GRNP (geno)toxicity screening. MRC-5 cells (pd19; ECACC catalogue number 05072101; lot: 13H007) were received from Sigma-Aldrich (Taufkirchen, Germany). MRC-5 cells exhibit fibroblast-like morphology, a predominantly normal 46, XY karyotype (evident in 56% of cells in the present study), and grow as an adherent monolayer. They are capable of approximately 45 population doublings before becoming senescent. Therefore, 19 population doublings in total were not exceeded. Under the culture conditions used, the mean population doubling time amounted to 24 h. Cells were propagated from the original pd19 aliquot, and the same working batch was used for validation purposes with a harmonized culture protocol by two different laboratories (Fraunhofer ITEM and the University of Aveiro). Cells were grown in Eagle’s Minimal Essential Medium (EMEM), alpha modification, supplemented with 2 mM L-Glutamine, 1% non-essential amino acids, 100 µg/mL streptomycin sulfate, 100 U penicillin G, sodium salt (PAA Laboratories GmbH, Cölbe, Germany) and 10% (*v*/*v*) FCS (same FCS batch in the two laboratories; Biochrom, Berlin, Germany). For cell propagation, cells were grown in T75 flasks at 37 °C and 5% CO_2_ in a humidified atmosphere using an incubator and were subcultured three times per week, when 70–80% confluent, using trypsin/EDTA (0.05%/0.02%). After thawing, cells were subcultured at least twice before the experiments, and then pre-cultured for 24 h before GRNP exposure. A complete medium exchange to the finally concentrated GRNP dispersions was performed at exposure start.

As an alternative to primary rat alveolar macrophages and to be independent of the use of animals for in vitro (geno)toxicity screening, the normal rat alveolar macrophage cell line NR8383 was included and evaluated regarding its predictivity for in vivo lung effects of GRNP. NR8383 cells were purchased from the American Type Culture Collection (ATTC, Manassas, VA, USA; AgC11x3A, NR8383.1]. BAL cells were originally isolated from a male Sprague-Dawley rat and subsequently transformed [30]. These cells grow as mixed cultures of adherent and suspension cells with a population doubling time of 32 h under the culture conditions used. NR8383 cells were grown in Kaighn’s modification of Ham’s F12 medium (F12-K; ATTC, Manassas, VA, USA) with 2 mM L-Glutamine, 1500 mg/L sodium hydrogen phosphate, 15% (*v*/*v*) FCS (Sigma-Aldrich, Germany), 100 µg/mL streptomycin sulfate, and 100 U penicillin G sodium salt (PAA Laboratories GmbH, Cölbe, Germany) on surface-activated culture vessels (TPP, Trasadingen, Switzerland) at 37 °C and 5% CO_2_ in a humidified atmosphere using an incubator. Cells received new culture medium twice a week by respecting the two cell fractions (adherent and suspension cells) and were subcultured once a week by incubation at room temperature for about 20 min with subsequent tapping. For experiments, NR8383 cells were used during passages 10 to 30 and were pre-cultured for 24 h before treatment with GRNP without medium exchange using double-concentrated dispersions. 

The murine macrophage-like cell line RAW 264.7 was purchased from the European Collection of Authenticated Cell Cultures (ECACC, Salisbury, UK). Cells were cultured in DMEM (Gibco, Life Technologies, Grand Island, NY, USA), supplemented with 10% (*v*/*v*) FCS (Gibco Life Technologies, Grand Island, NY, USA), 2 mM L-glutamine, 1% pen/strep (100 U/mL penicillin, 100 μg/mL streptomycin) and 2.5 μg/mL fungizone (Gibco, Life Technologies, Grand Island, NY, USA), in a humidified incubator at 37 °C and 5% CO_2_. Cell confluence and cell morphology were monitored frequently, and subculturing was performed when monolayers reached 75–80% of confluence.

#### 2.2.2. Cellular Uptake

Light microscopy served as a screening tool for evaluation of both cell density, cellular uptake (NR8383 cells; Figure S2) and cell morphology, as well as for estimation of density and homogeneity of the GRNP suspensions. For documentation, pictures were taken using a camera-equipped Nikon ECLIPSE TS 100 infinity-corrected inverse microscope.

To look for cellular uptake of GRNP by AM and MRC-5 cells, fluorescence-coupled darkfield microscopy (CytoViva, Auburn, AL, USA) was used. Therefore, cells were plated in one-well Nunc^TM^ Lab-Tek^TM^ II glass chamber slides (Thermo Fisher Scientific, Brunswick, Germany) at a density of 6 × 10^5^ and 2 × 10^5^ cells in 2 mL or 4 mL of cell culture medium, respectively. A GRNP concentration of 6.25 µg/cm^2^ was used for both cell types, as higher concentrations made it impossible to take meaningful pictures, because of the material overlay. For AM, the various materials were added as 2 mL of a double-concentrated dispersion without medium exchange. In contrast, for MRC-5 cells, GRNP were added after a medium exchange to the final mass-based concentration. Cells were subsequently incubated for 24 h, fixed with methanol/glacial acetic acid (3:1) and stained with 4′,6-diamidino-2-phenylindole (DAPI) to visualize cell nuclei. Finally, fluorescence-coupled darkfield microscopy was used to detect DAPI-stained cell nuclei via fluorescence and the GRNP materials via the darkfield microscopy unit.

#### 2.2.3. Cytotoxicity Screening

For cytotoxicity screening of GRNP, different endpoints, i.e., membrane damage, metabolic activity and cell proliferation, were investigated.

The influence of GRNP exposure on membrane integrity was investigated in all cell types by measuring lactate dehydrogenase (LDH) activity in culture supernatants after 24 h (depending on cell type) of incubation at 3.125, 6.25, 12.5, 25 and 50 µg/cm^2^. Concentrations were limited to a maximum of 50 µg/cm^2^ to avoid artificial results due to in vivo irrelevant concentrations. AM, NR8383, RAW 264.7 cells and MRC-5 cells were plated at a density of 1.5 (in 0.5 mL of culture medium), 0.56 (in 0.5 mL), 0.1 (in 0.5 mL) or 0.5 × 10^5^ cells (in 1 mL) per well of 24-well plates, respectively. Cells were pre-cultured for 24 h. For treatment with GRNP, double-concentrated stock dispersions (0.5 mL) were directly added to AM, NR8383 and RAW 264.7 cultures, whereas a medium exchange to 1 mL of the finally concentrated GRNP dispersions was performed for MRC-5 cells to eliminate persistent LDH activity resulting from subculturing. At the end of treatment, cell supernatants were carefully removed, transferred to 1.5-mL reaction tubes and centrifuged at 11,000× *g* for 30 min to eliminate residing particle material. LDH activity in culture supernatants was subsequently measured by transferring 100 µL of supernatant per well into optically clear 96-well flat-bottom microplates (3–4 technical replicates), adding 100 µL of the reaction mixture of the “Cytotoxicity Detection Kit” (Roche Diagnostics, Basel, Switzerland) and incubating of plates for 30 min at room temperature in the dark, before photometric measurement at 490 and 630 nm using an ELISA reader. Percent cytotoxicity was finally calculated using delta OD of the two wavelengths, subtracting the blank value and setting the result of Triton X-100-treated cells [1% (*v*/*v*) for 10–15 min] to 100% to finally calculate relative cytotoxicity.

For analysis of the relative increase in cell count (RICC) for estimating the impact of GRNP on cell proliferation, MRC-5 and NR8383 cells were seeded at a density of 0.56 × 10^5^ cells in 0.5 mL (NR8383) or 0.5 × 10^5^ cells in 1 mL (MRC-5) per well of 24-well plates and were pre-cultured for 24 h. Before treatment started, cell density was determined (starting cell count) using a Neubauer counting chamber. Cells were then treated with GRNP for 24 h. At the end of treatment, cells were detached, counted and RICC was calculated by subtracting the starting cell number from the cell count at the end of treatment (increase in cell count), dividing the results of the treated cells by the results of the negative controls and multiplying these by 100. 

Metabolic activity was investigated using the AlamarBlue^®^ test, based on the reduction of blue, oxidized resazurin to reduced pink resorufin. Cell culture medium without GRNP served as negative control and Triton X-100 [1% (*v*/*v*); 10–15 min] was used as a positive control (complete loss of viability). Additionally, cell culture medium without cells served as blank and GRNP background controls without cells (all materials and concentrations) were incubated in parallel to compensate for potential redox activity of GRNP or disturbance of photometric measurement by residual GRNP material. Incubation time amounted to 24 h. For AM and NR8383 cells, seeding and treatment of cells were performed as described above. After treatment, 10% (*v*/*v*) AlamarBlue^®^ reagent (Bio-Rad AbD Serotec GmbH, Puchheim, Germany) or AlamarBlue™ Cell Viability Reagent (ThermoFisher Scientific, Brunswick, Germany) was added to both the cell cultures or cell-free background controls for 2 or 8 h, respectively. Supernatants were finally sampled, centrifuged for 11,000× *g* for 30 min at 4 °C, 4 × 100 µL per sample were transferred to wells of a 96-well plate and were subsequently measured spectrophotometrically using a measured wavelength of 570 nm and a reference wavelength of 600 nm. In the cases of MRC-5 and RAW 264.7 cells, 7000 cells/well were seeded in 96-well plates, pre-cultured for 24 h and then treated with the GRNP dispersions. At the end of treatment, the medium was replaced by 100 µL of 10% (*v*/*v*) AlamarBlue^®^ reagent in cell culture medium, and cells were incubated for an additional 2 h before being directly measured. Like for the other cell types, GRNP background controls were used for background correction. After background correction, the percent reduction of AlamarBlue^®^, as an indicator of cell viability, was calculated according to the formula given by the providers. 

#### 2.2.4. Genotoxicity Screening

To look for induction of both DNA strand breaks and oxidative DNA damage, i.e., induction of the pre-mutagenic, oxidative DNA lesion 8 hydroxy-2′-deoxyguanosine (8-OHdG), the human 8-oxoguanine DNA N-glycosylase 1 (hOGG1)-modified alkaline comet assay [31] was performed with both AM and NR8383 cells. Therefore, AM (1.5 × 10^5^ cells) and NR8383 cells (5.6 × 10^4^) were plated in a volume of 0.5 mL in the respective 24-well plates and precultured for 24 h. Then, two cultures per treatment were incubated for 24 h with 25 µg/cm^2^ of the various GRNP samples, added to the culture as two-fold concentrated dispersions. KBrO_3_ (1 mM, 4 h) served as a methodological positive control, cell culture medium as vehicle control, and Al_2_O_3_ as a particle-like negative control. Al_2_O_3_ was dispersed in cell culture medium by ultrasonication for 15 min using a SONOREX Super RK 514 BH water bath (Bandelin electronics, Berlin, Germany). For gentle detachment, cells were placed on ice for at least 10 min at 4 °C. 

The following steps were performed as described previously [32]. After centrifugation, cells were resuspended in 80 µL of 0.75% (*w*/*v*) pre-heated low melting point agarose (LMA, peqGold No. 35-2010, Sigma-Aldrich, Taufkirchen, Germany) and applied to purpose-made slides with one roughened surface (Menzel-Gläser, Brunswick, Germany), pre-coated with normal melting agarose. After gelation at 4 °C, one additional layer of 100 µL of 0.75% LMA was applied. Slides were finally immersed in lysis solution [2.5 M NaCl, 100 mM Na_2_EDTA, 10 mM Tris-HCl, 8 g/L NaOH, 1% Triton-X100, 10% (*v*/*v*) dimethyl sulfoxide] and stored overnight at 4 °C. After cell lysis, slides were immersed in enzyme buffer (40 mM HEPES, 100 mM KCl, 0.5 mM Na_2_EDTA, 0.2 mg/mL bovine serum albumin, pH 8.0) and one of the two slides per treatment was incubated for 12 min at 37 °C in a humidified atmosphere with human hOGG1 by adding 0.16 U/gel of hOGG1 enzyme (New England BioLabs, Ipswich, MA, USA) in 100 µL of enzyme buffer to detect oxidative DNA base modifications. The other slide was incubated in parallel with enzyme buffer only. Electrophoresis was subsequently done using a pre-cooled electrophoresis platform filled with pre-cooled electrophoresis buffer (300 mM NaOH, 1 mM Na_2_EDTA, pH > 13). DNA was allowed to unwind for 20 min, before 24 V/300 mA were applied for 20 min. Finally, slides were neutralized with 0.4 M Tris-HCl (pH 7.4) and stained with ethidium bromide for at least 1 h. Slides were finally analyzed using an Axioskop fluorescence microscope (Carl Zeiss, Göttingen, Germany) and the Comet Assay III software (Perceptive Instruments, Bury St Edmunds, UK). The hOGG1-modified comet assay was also used to look for genotoxicity in bronchoalveolar lavage (BAL) cells from 5 animals per treatment group of the inhalation study after 28 days of recovery. Therefore, the cell number in BAL fluid was determined using a Neubauer counting chamber and 1.5 × 10^5^ BAL cells were then subjected to the hOGG1-modified alkaline comet assay as described above. As a follow-up approach, alkaline comet assays without enzyme modification were performed using the 0.5 × BMD30 and BMD30 concentrations for the different materials (see Section 2.2.5) to equalize cytotoxicity. 

BMD30 concentrations were also used for genotoxicity screening with RAW 264.7 cells. For RAW 264.7 cells, a slightly different comet assay protocol was used by the University of Aveiro. Briefly, cells were seeded in 6-well plates and incubated for 24 h at 37 °C with GFNs at BMD30 concentrations. Then, 20 µL of cell suspension (prepared in phosphate-buffered saline) were mixed with 70 µL of 1% low melting point agarose in distilled water. Eight drops with 6 µL of cell suspension were placed onto pre-coated slides (approximately 1000 cells). After solidification of agarose at 4 °C, slides were immersed in lysis solution (2.5 M NaCl, 0.1 M EDTA, 10 mM Tris, 1% Triton X-100, pH 10) for 1 h, at 4 °C. After lysis, slides were washed with cold buffer (0.1 M KCl, 0.5 mM EDTA, 40 mM HEPES, 0.2 mg/mL bovine serum albumin, pH 8) and incubated with enzyme buffer for 30 min at 37 °C in a humidified atmosphere. Slides were then immersed for 20 min in cold electrophoresis buffer (200 mM Na_2_EDTA, 10 mM NaOH, pH 13) to unwind DNA strands and expose alkali-labile sites. Electrophoresis was then conducted at 0.7 V/cm for 30 min at 4 °C, adjusting the current to 300 mA by raising or lowering the buffer level. Slides were finally neutralized with cold 0.4 M Tris buffer (pH 7.5) for 5 min and left to dry in the dark until staining and analysis. Prior to analysis, slides were hydrated with chilled distilled water for 30 min and stained for 20 min with propidium iodide (10 µg/mL). Slides were subsequently rinsed with distilled water to remove excess stain. DNA strand break induction was analyzed using a fluorescence microscope with 400× magnification and the Comet Assay IV software (Perceptive Instruments, Bury St Edmunds, UK).

Tail intensity (%) was selected by both laboratories as the main measure for DNA damage, as it represents the currently most accepted one. TI is, over a wide range, directly proportional to the number of DNA strand breaks induced. At least one hundred appropriately stained, non-overlapping nuclei were evaluated per treatment. Comets without heads were excluded. An increase in TI on the hOGG1-treated slides, as compared to the slides treated with enzyme buffer only, was indicative of the occurrence of the oxidative base lesion 8-OHdG. The mean of the single-cell data was calculated per slide. The means of three slides per treatment stemming from three independent experiments were finally subjected to statistical analysis.

#### 2.2.5. Calculation of Benchmark Dose

The benchmark dose (BMD) is a dose level estimated from the fitted dose–response curve, associated with a specified change in response, the benchmark response (BMR) [31]. This approach is applicable to all toxicological effects and estimates the dose that causes a low but measurable effect. It can be used as an alternative or in parallel with other dose descriptors (e.g., NOAEL). The benchmark dose lower confidence limit (BMDL) is the lower confidence bound of the BMD, while BMDU represents the upper confidence bound. The BMD approach can be used to derive a point of departure for further risk assessment [33,34,35]. In this study, the dose–response in vitro data were processed in order to calculate the BMD30, which represents the estimated dose corresponding to 30% of the cell viability reduction, using the PROAST software (version 38.9) as an R package [36]. BMD30 values for the different cells were then used to compare and rank the GRNP based on their cytotoxic potential (the response of 30% was selected due to the generally low cytotoxicity of GRNP, a parameter that allowed comparison of the materials tested).

### 2.3. In Vivo Validation

#### 2.3.1. Animals

Female Wistar rats [strain: Crl:WI (Han)] delivered by Charles River Deutschland Co. (Sulzfeld, Germany) were used for two animal experiments. The rats were acclimatized for 2 weeks in the animal facility and were aged 9 weeks at dosing. The animal studies had been approved by the competent authority (file # 33.19-42502-04-16/2286/LAVES, Oldenburg, Lower Saxony, Germany). For sacrifice, rats were anesthetized by intraperitoneal administration (0.1 mL per 100 g body weight) of sodium pentobarbital (Narcoren^®^) and exsanguinated by cutting the vena cava caudalis before preparation of the lungs.

#### 2.3.2. Dose Range Finding (DRF) Study (Intratracheal Instillation)

A DRF test was conducted to define an adequate dosing scheme for the final 28-day inhalation study. The rats were anaesthetized by CO_2_/O_2_ at 67/33 (*v*/*v*) for some seconds to perform intratracheal instillation of GRNP dispersions in physiological saline (0.9% NaCl). Concurrent controls were treated with vehicle only. Total particle doses were intratracheally instilled using two administrations on consecutive days (each day ½ of the total dose). Total GRNP and CB doses amounted to: P2 low (0.02 mg/lung); P2 high (0.2 mg/lung); P4 low (0.02 mg/lung); P4 high (0.2 mg/lung); Printex 90^®^ (0.2 mg/lung). Dose justification applied a space factor of 10 to mimic no lung overload in the low-dose groups and lung overload in the high-dose groups. 

For BAL, prepared lungs were lavaged with 0.9% NaCl, using two lavages with 4 mL each. In the pooled BAL fluid, leukocyte concentration was determined using a manual counting chamber, and two cytoslides were subsequently prepared for differential cell counting (macrophages, neutrophils, eosinophils, lymphocytes). After centrifugation of the BAL fluid, biochemical indicators relevant for the diagnosis of lung tissue damage were determined by standard methodologies in the supernatant [LDH activity, β-Glucuronidase (ß-Glu), total protein (TP)] [37]. The differential cell counts and enzyme/protein analyses were performed as described previously [38]. 

#### 2.3.3. 28-Day Nose-Only Inhalation Study

**Figure 2:**
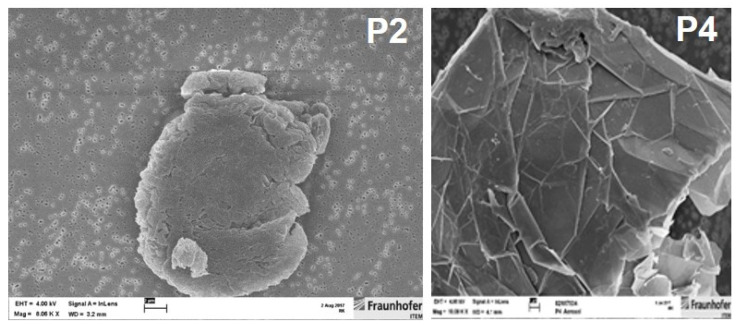
SEM photographs of aerosolized GRNP samples, i.e., P2 (left picture; SLG sample; magnification: 8000×) showing high and P4 (right picture; GNP sample; magnification: approx. 10,000×) showing low toxic potential in the in vitro screening with primary rat alveolar macrophages.

The two GRNP species P2 and P4 chosen for the 28-day nose-only inhalation study, based on in vitro screening results, were aerosolized using a dry dispersion system optimized for powdered substances and operated with pressurized air (high-pressure pneumatic disperser). For morphology of the aerosolized GRNP samples P2 and P4 see Figure 2. The disperser was fed under computerized control. The scattering light signal of an aerosol photometer was used to control the feed rate of the dispersion system in order to keep the aerosol concentration in the inhalation unit constant. Actual P2 and P4 concentrations were measured in the breathing zone of the animals. The aerosols were delivered to the rats in a flow-past nose-only inhalation exposure system. 

A 28-day nose-only inhalation test was conducted in accordance with [39]. Based on the in vitro results with AM, the two pristine graphene nanoplates showing the highest and lowest toxicity impact were selected for in vivo validation of the in vitro screening results. The SLG graphene factory series sample P2 was chosen for the test material with high adverse activity. It was preferred to the SLG sample P1, because of the affordable price for the considerable amount of test items needed for inhalation exposure (in the gram range). The GNP sample P4 was included as pristine graphene material with the lowest biological activity in AM in vitro. The test design, as depicted in Table 2, allowed for the direct comparison of a pristine SLG vs. a pristine GNP sample and estimation, amongst others, of the impact of graphene layers on potential lung toxicity. As a CB nanoparticle reference, Printex 90^®^ (=P8) completed the pattern of test items.

Because of the extremely light graphene materials (“mosquito behavior”), the precise measurement of the mass median aerodynamic diameter (MMAD) was challenging. In addition to the standard impactor-type Marple, an impactor of type Berner was also used. With the latter, the gravimetrical analysis is more reliable because higher absolute masses can be sampled on the filters. Measured MMAD values resulted in the ≤3 µm range, ensuring respirability of the aerosols.

Calculated doses (Multiple-Path Particle Dosimetry (MPPD) model) in the high-dose groups were similar to those administered in the intratracheal instillation DRF study: rat unisex: 0.2 L/min × 360 min/day × 20 days × 3.2 mg/m^3^ × 2.4% (deposition fraction) = 0.11 mg/lung (for comparison: high dose in DRF: 0.2 mg/lung -> actually 0.14 mg/lung following rapid clearance) [40]. For the exact dosing scheme and MMAD values, see Table 2.

The endpoints investigated in BAL fluid were differential cell counts, LDH, ß-Glu and TP, all of which were determined at day 1 and day 29 post-exposure using standard methods. In addition, the cytokines CINC-1 (a marker for macrophage recruitment) and osteopontin (an indicator of inflammation) were analyzed using specific ELISA kits (MesoScale Discovery-MSD; Multiplex) and concentrations of the stable thromboxane A_2_ (TXBA_2_; marker for acute lung injury) metabolite thromboxane B2 (TXB2) were measured by Dr. Dirk Schäfer (TalkingCells c.o. dysantec, Wiesbaden, Germany) using a highly specific competitive ELISA kit (Cayman Chemical, Ann Arbor, MI, USA).

Histopathology of the respiratory tract was performed for the lungs, including all five lung lobes and their main bronchi, the lung-associated lymph nodes (LALN) from the hilar region of the lung (LALN, mediastinal and tracheobronchial), trachea, larynx, pharynx and the nasal cavities including the nasal-associated lymphoid tissue (NALT). For histopathological examination, tissues were fixed in buffered formalin (10%) for up to one day, embedded in paraffin, sectioned, and stained with hematoxylin and eosin (HE). In addition, Masson trichrome staining was used for the detection of connective tissue production within the lung. The nasal cavity was decalcified following formalin fixation prior to paraffin embedding.

For cell cycle analysis in BAL, cell suspensions were centrifuged, the cell pellet fixed in 1 mL of 80% (*v*/*v*) ethanol and stored at −20 °C until analysis. Cell suspensions were then centrifuged (300× *g*, 5 min), resuspended in PBS and filtered through a nylon mesh into the test tubes before adding 50 µL of RNAse (Sigma-Aldrich, St. Louis, MO, USA) and 50 µL of propidium iodide (PI, ≥94%, Sigma-Aldrich, St. Louis, MO, USA). The samples were then incubated for 20 min at room temperature in the dark. PI-stained cells were finally analyzed on an Attune^®^ Acoustic Focusing Cytometer ((ThermoFisher Scientific, Brunswick, Germany)) and the percentages of cells at G0/G1, S and G2/M phases were determined using the FlowJo software (FlowJo LLC, Ashland, OR, USA).

#### 2.3.4. Statistics

Differences between groups in both the in vitro and in vivo experiments were considered statistically significant at *p* < 0.05. For in vivo endpoints, data were analyzed using analysis of variance. If the group means differed significantly in the analysis of variance, the means of the treated groups were initially compared with the means of the control groups using Dunnett’s test [41]. Finally, where indicated, the Student’s *t*-test for unpaired values, two-tailed, combined with normality (Shapiro-Wilk) and equal variance testing (Brown-Forsythe), was used for statistical analysis of most of the in vitro and in vivo endpoints (e.g., differential cell count, the liberation of TXB_2,_ hOGG1-modified alkaline comet assay, LDH release), comparing the treatment groups with the respective vehicle/negative control group using the SigmaPlot 14.0 software (Inpixon GmbH, Düsseldorf, Germany). To estimate the occurrence of oxidative DNA lesions, TI values from hOGG1-treated slides were compared with enzyme buffer-treated slides using the Student’s *t*-test for paired values, two-tailed. The statistical evaluation of the histopathological findings was done using the two-tailed Fisher test and statistical significance of flow cytometry data was calculated using the Dunnett’s test [41]. 

## 3. Results

### 3.1. GRNP Dispersion and Sedimentation Kinetics

Material morphology and dispersion grade are critical factors for cell interference in nanomaterial toxicology. For example, MWCNT were previously shown to induce malignant mesothelioma in rats after intraperitoneal administration, with morphology (more straight or curved MWCNT) determining both tumor incidence and the earliest appearance of tumors [42]. Ma-Hock et al. [22] further demonstrated in a 5-day inhalation study that carbon-based materials with different morphology and agglomeration state, i.e., MWCNT, CB, graphene and graphite oxide, behave very differently with regard to pro-inflammatory potential, with no effect for CB and GO, but pro-inflammatory effects for both MWCNT (highest adverse potential) and GNP. Even within the same group of carbon-based nanomaterials, i.e., MWCNT, Reamon-Büttner et al. [43] demonstrated a variable extent of in vitro effects such as membrane damage, inhibition of cell proliferation and induction of gamma-H2A.X pan-stained nuclei in human peritoneal mesothelial LP9 cells. While strong effects were observed for long and straight MWCNTs, no or nearly no effects were seen for tangled-type and milled MWCNTs, thus underlining the importance of material morphology.

**Figure 3:**
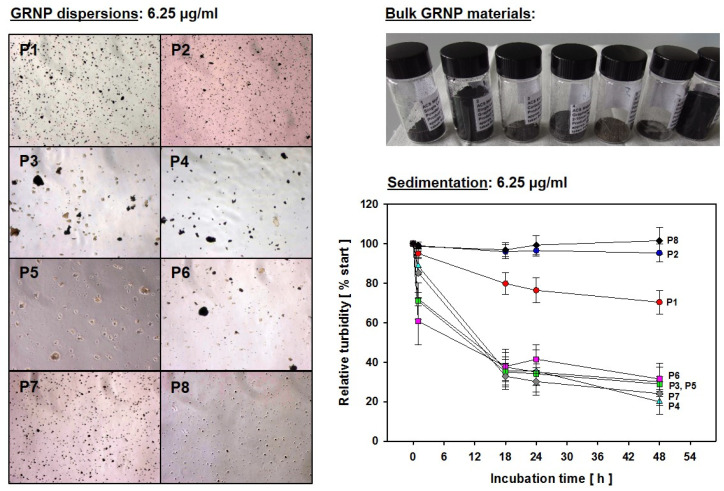
GRNP dispersions. Left: Light microscopic pictures of the different GRNP (P1–P8) dispersions at 6.25 µg/mL corresponding to the lowest concentration used for in vitro screening (3.125 µg/cm^2^). Right: Sedimentation kinetics for the various GRNP (P1–P8) at 6.25 µg/mL. GRNP dispersions were placed in semi-micro cuvettes and turbidity (OD600) was measured directly after material suspension (time point zero) and after 1, 18, 24 and 48 h of incubation at 37 °C in a water saturation atmosphere using an incubator. OD600 at time point zero was set to 100% and relative turbidity was calculated for the other time points. Data represent the means ± SD of three independent experiments.

In the present study, the dry GRNP powders showed macroscopically different colors and granularity (Figure 3) and exhibited diverse morphologies after dispersion in FCS-containing cell culture medium (Figure 1) as determined by SEM. The microscopic appearance comprised thin and in part transparent morphology for P2 (SLG) and P6 (graphite oxide), more sheet-like morphology for P3 (CG) and P4 (GNP), intensely folded morphology in the case of P1 (SLG) and P5 (GO) and more particle-like morphology for P7 (GNP). When estimating the homogeneity of dispersions and agglomeration state by light microscopy, P1 (SLG), P2 (SLG), P7 (GNP) and P8 (CB) demonstrated more homogenous and denser dispersions than P3 (CG), P4 (GNP), P5 (GO) and P6 (graphite oxide), which showed, irrespective of ultrasonication, diverse and in part big agglomerate/aggregate sizes (Figure 3). For P4, the observed heterogeneity in agglomerate sizes was in line with the particle size distribution given by the supplier, with a D10 of 13.56 µm, a D50 of 48.93 µm and a D90 of 122.2 µm. In addition, the sedimentation kinetics demonstrated marked differences for the various GRNP samples. While no or nearly no sedimentation was observed for P8 (no reduction in turbidity ± 6.71%) and P2 (4.7 ± 4.45%) after 48 h of incubation, P1 showed intermediate (29.6 ± 6.00%) and P7 (75.7 ± 5.85%) and P4 (80.0 ± 6.22%) highest sedimentation rates (Figure 3). Notably, the lowest sedimentation rates correlated with the highest specific surface, higher dispersion homogeneity and lowest biocompatibility in AM.

When evaluating the sedimentation kinetics, the question has to be raised whether differences in the extent of effects seen in the comparative in vitro screening experiments are in part a consequence of variable sedimentation rates and variable dispersion grades, leading to highly variable in vitro and intracellular material doses. Irrespective of the same mass-based concentrations, the different material dispersions might also considerably differ in nanoplate number, with, e.g., a higher nanoplate number for the two SLG species, leading to a completely black dispersion with the highest concentration used (50 µg/cm^2^). A clear definition of in vitro doses for non-particle-like nanomaterials, i.e., nanoplates and fibers, thus poses a real challenge and should be an important topic for future research along with the quantification of intracellular material in the field of GRNP materials.

### 3.2. In Vitro Screening

#### 3.2.1. Cytotoxicity Screening

After evaluating different dispersion methods for submerse GRNP exposure of cells, comparative cytotoxicity screening was performed using different lung-relevant cell models, which covered three different species (rat, mouse and human), primary cells and immortalized cell lines and two different cell types, i.e., rat alveolar macrophages/macrophage-like cells and fetal lung fibroblasts, to evaluate the predictivity of the cell models for the in vivo situation after lung exposure.

Cytotoxicity screening was started with the two lung-relevant, normal cell models, i.e., primary rat alveolar macrophages (AM) and human MRC-5 lung fibroblasts (investigated in two independent laboratories), to initially exclude an impact of cell immortalization. Cells were incubated for 24 h with the different GRNP samples at 3.125, 6.25, 12.5, 25 and 50 µg/cm^2^ to enable the calculation of BMD30 values for the final ranking of material adversity. Membrane damage (LDH release), metabolic activity (AlamarBlue^®^ assay), and RICC (cell counts; MRC-5 cells) were chosen as endpoints because of ease of measurement and, thus, good applicability for in vitro screening approaches. LDH activity, furthermore, represented a direct correlate to the in vivo investigations.

**Figure 4:**
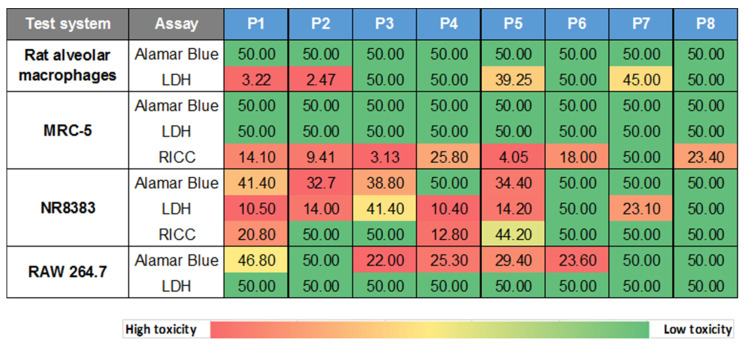
Heatmap of in vitro cytotoxicity screening results. The cytotoxic potential of GRNP was screened in primary rat alveolar macrophages (AM), human MRC-5 lung fibroblasts, the rat alveolar macrophage cell line NR8383 and the murine macrophage-like cell line RAW 264.7. Cells were incubated for 24 h with the various GRNP samples and membrane damage (LDH release), metabolic activity (AlamarBlue^®^ test) and cell proliferation (RICC; MRC-5 and NR8383 cells) were determined. Numbers given represent BMD30 values [µg/cm^2^], calculated from three independent experiments with five concentrations (3.125, 6.25, 12.5, 25 and 50 µg/cm^2^) each. A value of 50 is given where the final BMD30 value was calculated to be greater than 50 µg/cm^2^.

In MRC-5 cells, there was no GRNP sample that reproducibly mediated at least a 30% increase in membrane damage or reduction in metabolic activity in MRC-5 fibroblasts after 24 h of incubation in the concentration range tested (see BMD30 values in Figure 4). Despite the replacement of the GRNP dispersions before the final addition of the AlamarBlue^®^ reagent and the use of cell-free background GRNP controls, however, an artificial reduction of the AlamarBlue^®^ reagent seemed to occur, a phenomenon that was also observed in methylthiazoyldiphenyl-tetrazolium bromide (MTT) assays with GO and graphene sheets and human skin fibroblasts [44]. The resulting higher reduction values as compared to the negative control might mask cytotoxic effects. Notably, a proliferation-inhibiting effect of the GRNP materials was observed in MRC-5 cells using RICC and, thus, cell counts as an endpoint. The decrease in RICC in MRC-5 cells seemed to correlate with GRNP thickness, as P5, P3, P2 and P1 demonstrated the lowest BMD30 values (thickness between 0.6 and 1.2 nm), followed by P6 (1–3 nm), P4 (2–10 nm) and P7 (3 nm), and might indicate direct mechanical interference with the cytoskeleton and the mitotic apparatus of the cells, as discussed previously for induction of micronuclei by two types of pristine graphene nanoplatelets in THP-1 cells [45].

In contrast to the MRC-5 lung fibroblasts, both SLG materials (P1 and P2) with the highest specific surface and lowest sedimentation rates mediated strong, concentration-dependent membrane damage in primary rat alveolar macrophages (AM). BMD30 values amounted to as low as 3.22 and 2.47 µg/cm^2^. Some increase in LDH activity was also observed for P5 (GO) and P7 (GNP), with remarkably higher BMD30 values of 39.25 and 45 µg/cm^2^, respectively. All other GRNP samples, including P4 (later used for in vivo validation) as well as CB (P8) samples, exhibited no relevant membrane-damaging effect, as judged by BMD30 values equal to or below 50 µg/cm^2^ (Figure 4). The final ranking was, thus, P2 (SLG) > P1 (SLG) > P5 (GO) > P7 (GNP). 

**Figure 5:**
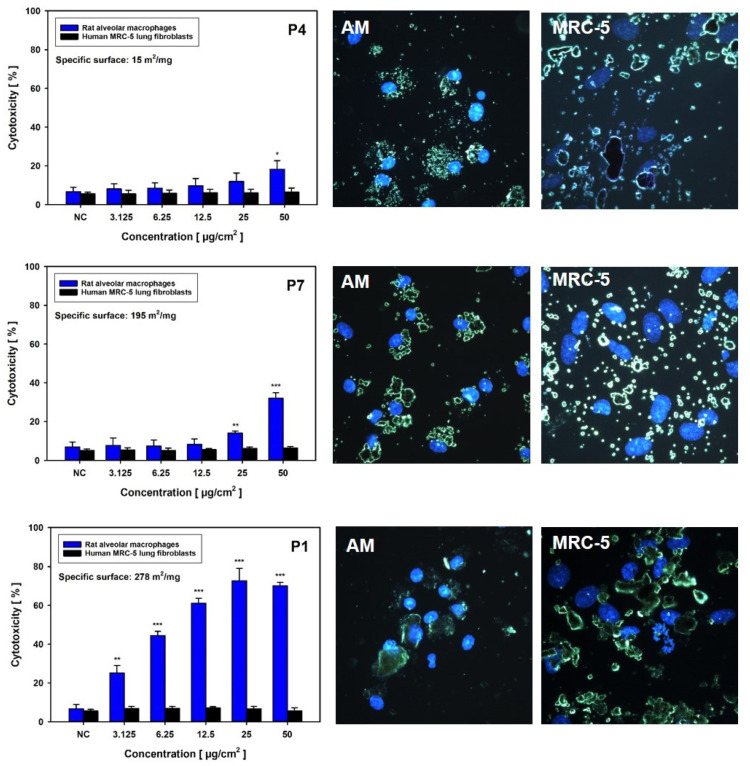
Material-dependent induction of membrane damage and cellular uptake in rat alveolar macrophages (AM; blue bars) and human MRC-5 lung fibroblasts (black bars). Cells were incubated without (NC) or with the given concentrations of P4, P7 or P1 for 24 h, with subsequent measurement of LDH activity in culture supernatants. Data represent arithmetic means ± SD of three independent experiments. */**/*** Statistically significant difference from NC: *p* ≤ 0.05, *p* ≤ 0.01 and *p* ≤ 0.001, respectively. On the right-hand side, fluorescence-coupled darkfield microscopy pictures are given for both cell types. Cells were incubated for 24 h with P4, P7 or P1 at 6.25 µg/cm^2^. Cell nuclei were subsequently stained with DAPI.

Notably, AM seemed to be finally predictive of the in vivo situation when considering LDH release. The LDH data for AM were clearly in line with subsequent in vivo validation, as a strong effect was noted for P2, but no effect for P4. Disturbance of the cell membrane in AM by GRNP was, furthermore, shown to be time-dependent, with a statistically significant, small effect for P2 after 4 h at 50 µg/cm^2^ (19.7 ± 6.98% cytotoxicity; 72.3 ± 8.32% cytotoxicity after 24 h; Figure S1) only, and there seemed to be some correlation with the specific surface area of the GRNP samples, as a ranking of P1 (BET: 278 m^2^/g) > P7 (BET: 195 m^2^/g) > P4 (BET: 15 m^2^/g) was obvious (Figure 5). Interestingly, P3 (CG) seemed to quench LDH activity under certain conditions. Additional characteristics mediating the stronger membrane-disturbing effect of the SLG samples, compared to the other GRNP, should be further investigated but might comprise a higher particle number, better dispersion or different morphology, with potentially sharper edges, which could mediate membrane damage in the case of cell movements or cell overload or direct cutting of cell membranes.

To investigate the reason for the absence of LDH release in the MRC-5 model, GRNP uptake was determined after 24 h of incubation using fluorescence-coupled darkfield microscopy. Fluorescence-coupled darkfield microscopy showed that all GRNP materials were taken up by AM. The cells were more or less tightly packed with GRNP material, located in direct proximity to the cell nucleus (Figure 5). In contrast, when evaluating GRNP uptake in MRC-5 cells, the materials seemed predominantly attached to the cell surface but were not taken up to a marked extent. No obvious differences were observable between the different test materials, except for different cell morphologies and agglomerate/aggregate sizes.

As described for MRC-5 cells, GRNP samples mediated an unspecific reduction of the AlamarBlue^®^ reagent in AM, however, to a lesser extent. This was perhaps based on a different assay protocol with the measurement of the supernatant after centrifugation and not a direct measurement of the cell culture plates. A very slight tendency towards a concentration-dependent decrease in reduced AlamarBlue^®^ was only observed for the SLG sample P2, with 40.6 ± 6.03% at 3.125 µg/cm^2^ as the lowest concentration and 29.4 ± 6.10% at 50 µg/cm^2^ as the highest concentration. A very slight tendency was also observed for P1 and P7, but none of these reached statistical significance, compared to 36.2 ± 3.01% for the negative control. No effect was evident for P4 and all other GRNP.

In further experiments, cytotoxicity of the GRNP panel was investigated in NR8383 and RAW 264.7 cells as immortalized macrophage models to look for an animal-independent, generally available screening model. However, in vivo prediction seemed to be limited for the two cell types in the present study, based on cytotoxicity screening. RAW 264.7 cells showed no induction of membrane damage by the GRNP samples (Figure 4 and Figure 5), and in the AlamarBlue^®^ assay, the GNP sample P4, but not the in vivo active P2 (SLG) sample mediated a decrease in viability (Figure 4). However, this reciprocal ranking, compared to AM, was in line with the subsequent uptake experiment with RAW 264.7 cells, which demonstrated a significantly higher uptake for P4 than for P2. NR8383 cells, in fact, demonstrated a slightly lower BMD30 value for P2 than for P4 in the AlamarBlue^®^ assay, but irrespective of a membrane-damaging potential of GRNP in NR8383 cells, the LDH assay demonstrated comparable BMD30 values for P2 and P4 and, additionally, a reciprocal activity ranking based on RICC results, making predictions difficult (Figure 4).

The results from AM were in line with previous reports on different cell types, pointing to higher cytotoxicity of pristine graphene and, in particular, SLG or FLG graphene, compared to GO (single-layer), graphite or surface-modified graphene nanoplates. In a previous study, Hinzmann et al. evaluated the impact of different GRNP on the viability of the glioblastoma cell line U87. Notably, as determined by trypan blue exclusion, the graphene sample demonstrated higher cytotoxicity than GO or graphite [46]. Using human bronchial epithelial Beas2B cells and 24 h of incubation, Chatterjee et al., furthermore, showed higher toxicity of pristine FLG (<4 layers) than of GO (single-layer) and CG using the endpoints of colony formation and metabolic activity [47]. Higher cytotoxicity of pristine graphene versus CG was thought to depend on hydrophobic interactions of pristine graphene with the cell membrane, with subsequent intracellular ROS generation and induction of apoptosis, whereas the more hydrophilic CG was taken up by cells without inducing marked cytotoxic effects [48]. The higher membrane-damaging activity of the SLG versus the GNP samples in AM might be based on the number of graphene layers, with higher stiffness and consequently lower activity of the GNP, as previously discussed by Muzi et al. to explain the very low cytotoxic potential of two multi-layer graphene samples in RAW 264.7 cells [49].

#### 3.2.2. Genotoxicity Screening

In the initial cytotoxicity screening, the different macrophage models seemed to represent the more sensitive screening models when compared with human MRC-5 lung fibroblasts, most likely based on limited uptake of GRNP by fibroblasts (Figure 5 and Figure S2). Therefore, genotoxicity screening experiments were performed with the macrophage models only. There are only limited in vitro genotoxicity data, in particular for pristine graphene nanoplates in lung-relevant cell types, and potential modes of action are still under discussion, comprising direct DNA damage by entry into the cell nucleus and cutting or binding of DNA, amongst others, but also indirect DNA damage by the generation of reactive oxygen species by pristine graphene [13,48].

**Figure 6:**
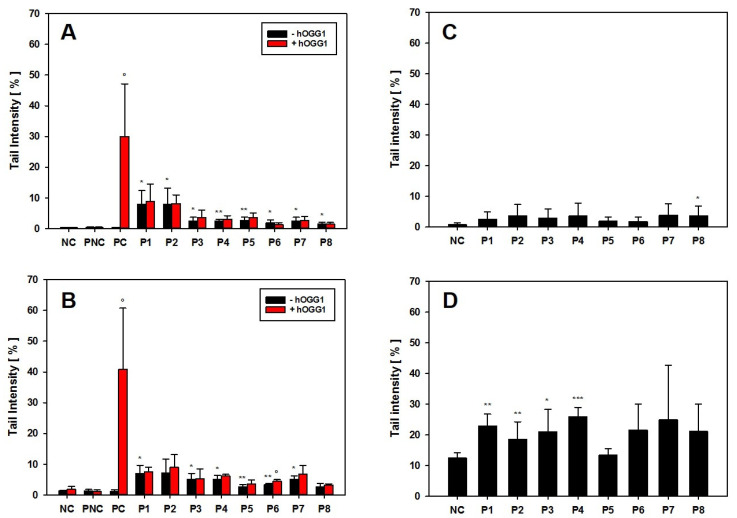
Genotoxicity screening of GRNP materials in AM, NR8383 cells and RAW 264.7 cells. (**A**,**B**) hOGG1-modified alkaline comet assay. AM (**A**) or NR8383 cells (**B**) were incubated for 24 h without (negative control, NC) or with 25 µg/cm^2^ of P1–P8. Al_2_O_3_ served as particle-like negative control (PNC) and KBrO_3_ was used as positive methodological control. Data represent the arithmetic mean tail intensities (TI) ± SD of three independent experiments. Increase in TI on slides with hOGG1 treatment, compared to the respective slides without hOGG1, is indicative of oxidative DNA lesions. (**B**,**C**) Alkaline comet assay. AM (**C**) or RAW 264.7 cells were incubated for 24 h with or without the calculated BMD30 concentrations of P1–P8. Data represent the arithmetic mean TI ± SD of three independent experiments. For all experiments, the arithmetic mean was used as summarizing measure for the single-cell data on slides. */**/*** Statistically significant difference from PNC (**A**,**B**) or the respective NC (**C**,**D**): *p* ≤ 0.05, *p* ≤ 0.01 and *p* ≤ 0.001, respectively; Student’s *t*-test for unpaired values, two-tailed. ° Statistically significant difference from slides without hOGG1 treatment: *p* ≤ 0.05; Student’s *t*-test for paired values, two-tailed.

For genotoxicity screening, the hOGG1-modified comet assay with AM and NR8383 cells was used to look for both DNA strand break induction and oxidative DNA damage, i.e., 8-OHdG as a pre-mutagenic DNA lesion. The induction of reactive oxygen species with subsequent oxidative DNA damage has often been discussed as an indirect mode of action for nanomaterials. AM and NR8383 cells were incubated for 24 h with 25 µg/cm^2^ with either the different GRNP, culture medium only (negative control), or Al_2_O_3_ (particle-like negative control). KBrO_3_ served as technical positive control for both DNA strand break induction and oxidative DNA damage. Slight, but statistically significant induction of DNA strand breaks was observed in AM for all GRNP samples (Figure 6A) compared to the medium (mean TI: 0.29 ± 0.076%) and the particle-like negative control (Al_2_O_3_; mean TI: 0.43 ± 0.159%). The SLG samples P1 (mean TI: 7.93 ± 4.424%) and P2 (mean TI: 7.89 ± 5.351%), as the most active GRNP materials in cytotoxicity screening, mediated comparable, approximately 4.5-fold higher mean TI values than P8 (mean TI: 1.56 ± 0.590%) and P6 (1.79 ± 1.081%), as the GFN samples with the lowest activity (Figure 6A). In the present study, the arithmetic mean was used as summarizing measure for the cell nuclei analyzed per slide to consider the more bimodal than normal distribution of induced DNA damage in the case of particulate test items, and to enhance sensitivity. Like in AM, the SLG samples P1 and P2 mediated the highest effects in NR8383 cells (Figure 6B).

Higher genotoxic activity of pristine graphene (<4 layers) than of GO (single-layer) at 10 and 50 µg/mL was described previously in lung-relevant Beas2B cells after 24 h of incubation using the alkaline comet assay [3]. Additionally, Hinzmann et al. investigated the induction of DNA strand breaks in U87 cells after 24 h of incubation with subsequent activity ranking of pristine graphene > graphite > GO. Unfortunately, no nanoplate dimensions were given to estimate whether pristine SLG, FLG or GNP were used [46]. The results of the hOGG1-modified comet assay were finally in line with the comet assay data from the BAL cells of the 28-day inhalation study on day 29 post-exposure, with higher induction of DNA strand breaks for P2 than for P4.

None of the GRNP samples induced oxidative DNA lesions in both AM and NR8383 cells, as there were no higher mean TI values for the slides incubated with the DNA repair enzyme hOGG1 (specific for oxidative DNA lesions, i.e., 8-OHdG), compared to the slides incubated with enzyme buffer only. A false-negative result could be excluded based on the strong effect of KBrO_3_ as a specific positive control for induction of 8-OHdG (Figure 6A,B). However, a trend toward oxidative DNA damage was noted in the BAL cells in the 28-day inhalation study. 

As the comet assay is supposed to be sensitive to unspecific effects based on excessive cytotoxicity, follow-up experiments were performed using the BMD30 concentrations calculated from LDH release data to equalize for cytotoxicity. In these experiments, all GRNP samples mediated nearly the same effects, when compared to the respective negative controls in both AM and RAW 264.7 cells (Figure 6C,D). In AM, no GRNP material reached statistical significance, except for P8 (CB). In RAW 264.7 cells, higher TI values were observed for both the negative control and the GRNP-treated cultures. Statistically significant higher TI values were observed for P1–P4. P5 (GO) demonstrated the lowest TI values in both cell types, in RAW 264.7 cells, even comparable to the negative control. The outcome of the control experiments with AM and RAW 264.7 cells, thus, might indicate that GRNP-mediated DNA strand break induction might be of a more unspecific nature, based on cytotoxicity, and less likely due to direct genotoxicity. Alternatively, alignment of the genotoxic potential might indicate an adaptation of particle numbers.

### 3.3. In Vivo Validation

From the initial in vitro (geno)toxicity screening of various GRNP it was concluded that AM with the endpoints LDH release and DNA strand break induction might represent a fair and not hypersensitive screening model to predict in vivo lung toxicity of GRNP. To confirm this assumption, in vivo validation was performed with one of the two pristine graphene samples that were most active in vitro in AM, i.e., P2 (SLG; sufficient material available) and P4 (GNP) as the least biologically active pristine graphene sample. Two pristine graphene nanoplates were finally chosen for the 28-day inhalation study, not only due to their strikingly different cytotoxicity in AM in vitro, but also because in vivo lung toxicity data are still sparse for this subgroup of GRNP. This choice, furthermore, enabled comparison of graphene nanoplates with single- (P2) and multi-layer morphology (P4) and thus, different thicknesses (1–5 atomic layers versus 2–10 nm) and resulting stiffness, and investigation of GRNP materials with markedly varying specific surface areas (620 versus 15 m^2^/mg).

#### 3.3.1. Dose-Range Finding Test (DRF) with Instillation into Rat Lungs

**Figure 7:**
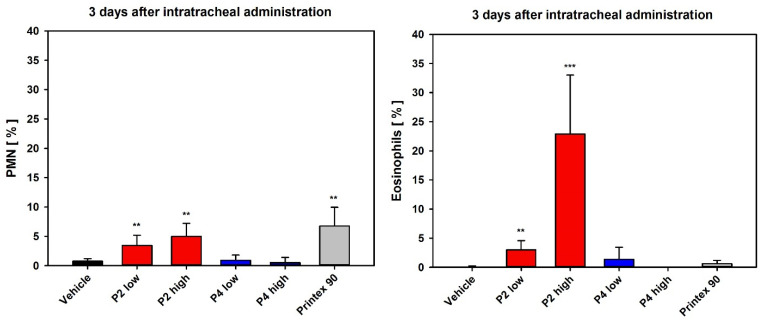
Differential cell counts 3 days after intratracheal instillation of P2 and P4. Note the high eosinophilic percentage, indicating a specific immunological response. Depicted are relative PMN and eosinophil numbers. Data represent means ± SD of four to five animals per group. **/*** Statistically significant difference from vehicle control (vehicle): *p* ≤ 0.01 or *p* ≤ 0.001, respectively; Student’s *t*-test for unpaired values, two-tailed.

In the DRF test with intratracheal instillation, P2 showed a very slight yet statistically significant increase in inflammatory lung effects, i.e., an increase in polymorphonuclear neutrophils (PMN; unspecific response) in the low-dose group 3 days after instillation, and a slightly higher and statistically significant increase in PMN values in the high-dose group. In contrast, P4 mediated no significant increase in PMN influx. Notably, in the differential cell counts, strong induction of eosinophils was observed in BAL fluid of the P2 high-dose group, which is normally not detected in the rat model after particle exposure. This pointed to a specific inflammatory response, whereas the PMN showed markedly lower values (Figure 7). In the P2 low-dose group, enzyme activities of LDH and ß-Glu, as well as TP, were not significantly increased, whereas in the P2 high-dose group, a statistically significant increase occurred. This was also observed in the P4 high-dose group, however, to a lower degree (Table S1).

#### 3.3.2. 28-Day Rat Inhalation Test

**Figure 8:**
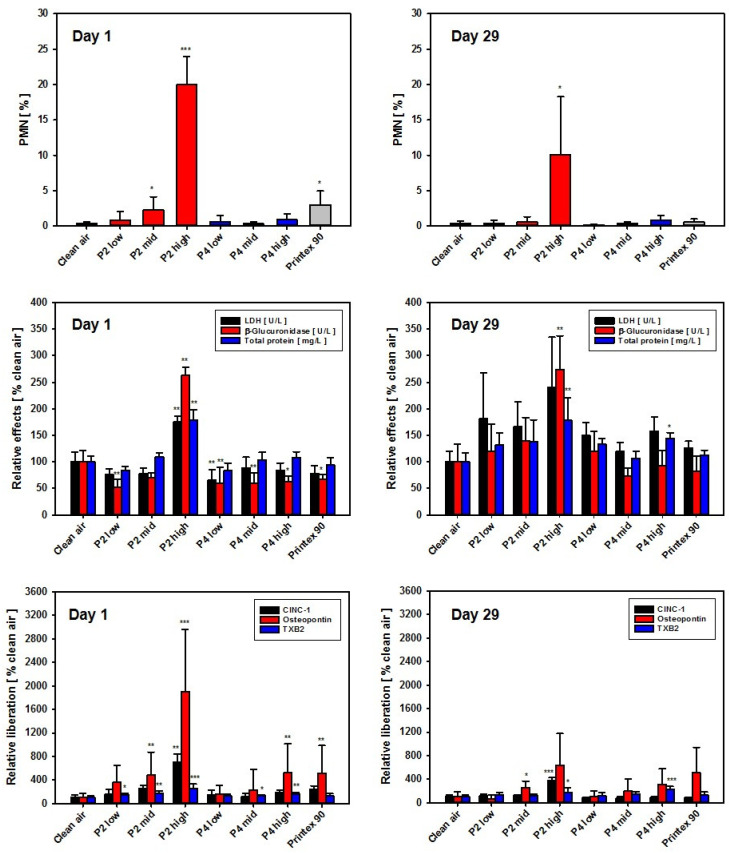
Results of BALF analysis at day 1 (left) and day 29 (right; 28 days of recovery) post-exposure. Depicted from differential cell counts are relative PMN numbers only. Top: PMN [% of total leukocytes]; middle: LDH activity, β-Glu activity, and total protein in BAL [relative to clean air controls set to 100%]; bottom: CINC-1, osteopontin and TXB_2_ concentrations in BAL [relative to clean air controls set to 100%]. Data represent the means ± SD of five animals per group. */**/*** Statistically significant difference from negative control (clean air): *p* ≤ 0.05 or *p* ≤ 0.01 or *p* ≤ 0.001, respectively; Student’s *t*-test for unpaired values, two-tailed.

In the 28-day nose-only inhalation test, BAL fluid analysis in the P2 (SLG) low- and mid-dose groups revealed PMN values at the control level, whereas in the P2 high-dose group, an induction of moderate inflammation was noted. After recovery, the latter group still showed slight inflammation. All three P4 (GNP) groups, as well as the carbon black group, showed PMN values at clean air levels. Surprisingly, induction of eosinophil recruitment, as observed in the DRF study after intratracheal instillation, was not observed after inhalation. In the P2 high-dose group, but not in the P4 high-dose group, LDH levels were significantly increased, mirroring the results observed in the respective in vitro assay with AM (Figure 8).

In the P2 low- and mid-dose groups, BAL fluid analysis showed biochemical results close to clean air control levels. In the high-dose group, however, all three parameters (LDH, β-Glu and TP) were statistically significantly increased. All other groups, i.e., all P4 (GNP) dose groups as well as the Printex 90 (CB) group, remained at control levels. Following recovery, with the exception of the P2 high-dose group (SLG), all biochemical endpoints and PMN percentages, as well as absolute numbers in BAL fluid, had returned to clean air levels. In the P2 high-dose group, however, the PMN moiety still remained at approx. 10% and the biochemical endpoints β-Glu and TP, as well as the absolute PMN numbers, were still significantly increased (Figure 8 and Figure S4).

At day 1 post-exposure, CINC-1 showed clear dose-dependence. When comparing the high-dose groups of P2 and P4, the latter showed a 3.7-fold lower value. Osteopontin results mirrored this finding. At day 29 post-exposure, following a 4-week recovery period, the dose dependence of CINC-1 levels continued at reduced values in the P2 groups, whereas all P4 groups returned to control group values. For osteopontin, increased values were detected in the mid- and high-dose groups of P2 and P4, with P2 again exhibiting maximum values (Figure 8).

As an additional endpoint for the 28-day nose-only inhalation study, the content of TXB_2_ as a stable metabolite of the eicosanoid TXA_2_ was measured in BAL fluid. A concentration-dependent, highly statistically significant increase in TXB_2_ was noted in BAL fluid of the P2 high-dose group at day 1 post-exposure, amounting to about 3-fold higher levels than in the vehicle controls. In contrast, P4 only mediated a very small increase. After 29 days of recovery, the P2 and P4 high-dose groups mediated a comparable, about 2-fold increase in TXB_2_, as compared to the saline control (Figure 8, bottom graphs). TXB_2_ seemed to represent a promising eicosanoid marker with regard to early detection and differentiation of inflammatory effects of different GRNP in the rat lung. Notably, concentration-dependent induction of TXB_2_ liberation by GRNP treatment for 24 h was also observed in preliminary in vitro experiments with NR8383 cells (Figure S3). Increased TXB_2_ levels were only seen in pristine graphene nanoplate samples (P1, P2, P4 and P7) and P5 (GO), but were nearly absent for P3 (CG), P6 (graphite oxide) and P8 (CB).

**Figure 9:**
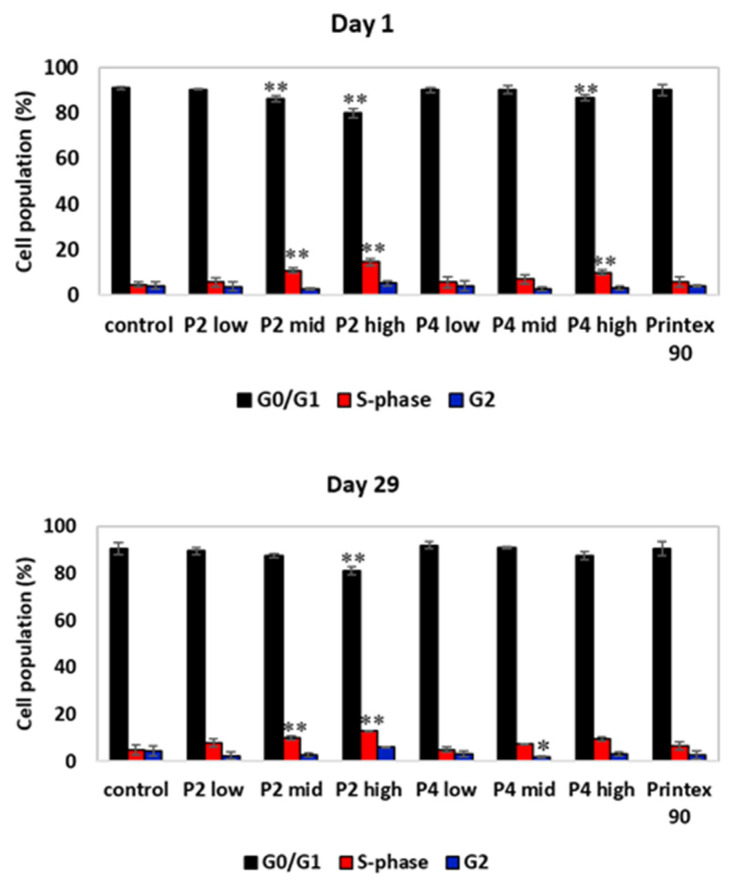
Cell cycle analysis of BAL cells on day 1 and day 29 post-exposure of the 28-day nose-only inhalation study. The distribution of cells in each of the cell cycle phases was based on cell DNA content by staining with propidium iodide. The percentage of cells at each of the cell cycle phases was determined using the FlowJo software. */** Statistically significant difference from control at *p* ≤ 0.05, *p* ≤ 0.001, respectively; Dunnett’s test.

Cell cycle analysis with BAL cells demonstrated a decrease in the percentage of cells at G0/G1 with increasing P2 dose, which for the P2 mid- and P2 high-dose groups was followed by an increase in the percentage of cells at S-phase (Figure 9). This cell cycle profile for P2-exposed animals was mostly maintained after the 29-day recovery period, suggesting that P2 treatment can induce a prolonged S-phase arrest. Exposure to P4 at the high dose also induced a decrease in the percentage of cells at G0/G1 and an increase in the percentage of cells at the S-phase, which was recovered after 29 days of recovery. At this time point, however, a decrease in the percentage of cells at G2 for the P4 mid-dose group was detected. Previous studies have shown that graphene derivates may induce alterations in the cell cycle dynamics. For instance, Hashemi et al. [50] found that nano- and micron-sized GO triggered the cell cycle of fibroblasts and induced an arrest of cells at the S-phase. Furthermore, Wang et al. [51] showed an S-phase block in HEK293T, MCF-7, A549, and HepG2 cells exposed to GO-PEG-PEI. Proper progression through cell division is assured by cell cycle checkpoints that keep the cell from progressing to the next phase of the cell cycle before the prior phase has been completed. The S-phase checkpoint is activated under conditions of threatened DNA replication, such as DNA damage. This activation results in S-phase arrest, inhibiting DNA replication and promoting DNA repair mechanisms, checking the fidelity of DNA replication. Additionally, an increase in the coefficient of variation (CV) of the G0/G1 peak was observed in the P2 high-dose and P8 (CB) groups, which may reflect clastogenic effects (Figure S5).

**Figure 10:**
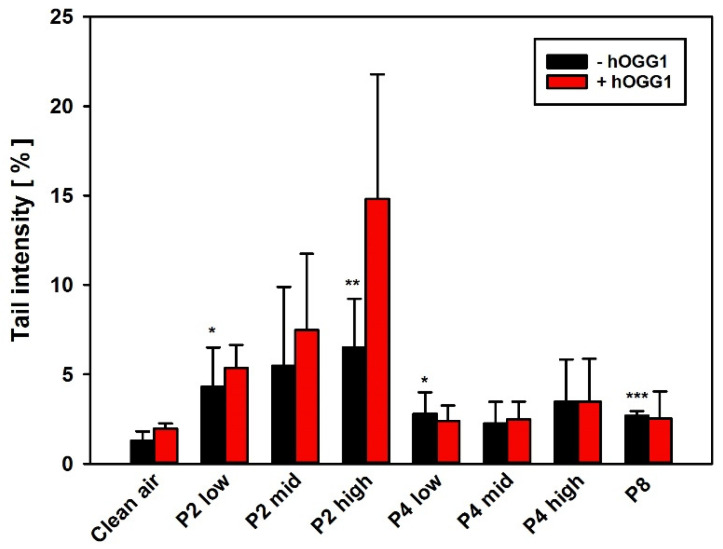
The hOGG1-modified comet assay with BAL cells on day 29 after the last inhalation. AM were isolated by alveolar lavage and subsequently analyzed regarding induction of DNS strand breaks and oxidative DNA lesions. Data represent the arithmetic mean tail intensities (TI) ± SD of 5 animals per treatment group, using the arithmetic mean as summarizing measure for the single-cell data on slides. An increase in TI on slides with hOGG1 treatment, as compared to the respective slides without hOGG1 incubation, is indicative of induction of oxidative DNA lesions, i.e., 8-OHdG. */**/*** Statistically significant difference from clean air control: *p* ≤ 0.05, *p* ≤ 0.001 and *p* ≤ 0.001, respectively; Student’s *t*-test for unpaired values, two-sided.

In the hOGG1-modified comet assay with BAL cells, a concentration-dependent induction of DNA strand breaks for P2, as well as a tendency towards oxidative DNA damage was observed. In contrast, P4- and P8-treated animals demonstrated markedly lower induction of DNA strand breaks after 29 days of recovery (Figure 10). This was in line with the results of the cell cycle analysis with BAL cells (Figure 9 and Figure S5) and the observation of still slight inflammatory effects in the P2 high-dose group after 29 days post-exposure, but the absence of marked inflammation in P4-treated animals and the P8-treated particle-like CB reference, as judged, in particular, by PMN, lymphocyte numbers, enzyme activities and TP in BAL fluid (Figure 8 and Figure S4). A tendency toward the 8-OHdG formation and, thus, an increase in oxidative DNA damage was most likely linked to P2-induced secondary genotoxicity by the inflammation-dependent generation of reactive oxygen species.

Histopathological examination of the respiratory tract revealed findings related to the inhalation of the GRNP samples P2 (SLG), P4 (GNP) and P8 (CB particle-like reference). Findings were observed in the lung, the lung-associated lymph nodes and the nasal cavity. In the nasal cavity, eosinophilic globules were seen in a multifocal pattern in the olfactory epithelium, most prominently in the P2 high-dose group, with a slight increase during the 29-day recovery period (Table 3 and Table 4).

**Figure 11:**
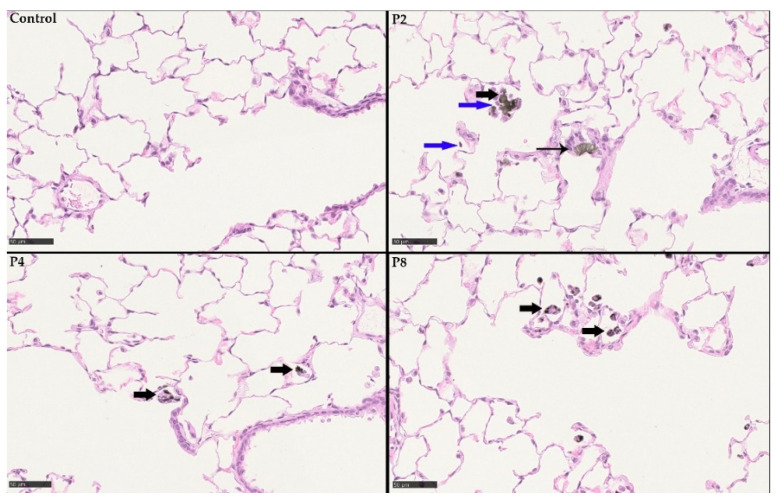
Histopathological findings at day 1 post-exposure following 28 days of inhalation. Control without any histopathological findings. P2 (SLG) high-dose group with particle-laden macrophages (bold black arrows), particle-laden giant cells (syncytia; black arrow) and infiltration of granulocytes (blue arrow). P4 (GNP) high-dose group with particle-laden macrophages (bold black arrows). P8 (CB) also with particle-laden macrophages (bold black arrows).

**Figure 12:**
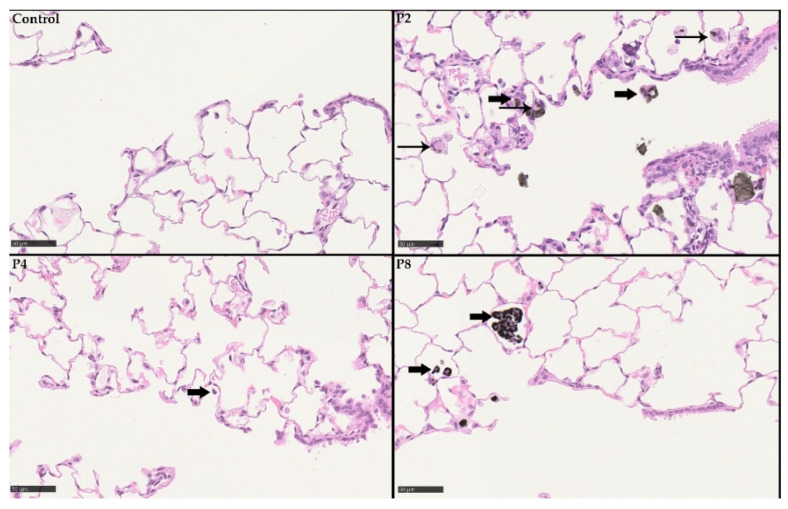
Histopathological findings at day 29 post-exposure following 28 days of inhalation. Control without any histopathological findings. P2 (SLG) high-dose group with particle-laden macrophages (bold black arrows) and particle-laden giant cells (syncytia; black arrow). P4 (GNP) high-dose group with particle-laden macrophages (bold black arrows). P8 (CB) also with particle-laden macrophages (bold black arrows).

The lung showed the dose-dependent occurrence of particle-laden macrophages in the alveoli, lung interstitium (Figure 11 and Figure 12), and the bronchus-associated lymphoid tissue (BALT). Notably, some macrophages fused time-dependently to giant cells (syncytia), which were visible mainly in the P4 high-dose group (Figure 11 and Figure 12 and Table 3 and Table 4). Additionally, alveolar infiltration of granulocytes occurred in the P2 high-dose group on day 1 post-exposure after 28 days of inhalation, declining during the recovery period (Table 3 and Table 4). In the lung-associated lymph nodes, accumulation of particle-laden macrophages was found in the P8 carbon black-treated group at day 1 post-exposure after 28 days of inhalation, as well as in the P2 high-dose and the P8 groups after 29 days of recovery (Table 3 and Table 4). A histopathological finding that was interpreted as adverse was alveolar infiltration of granulocytes, which was observed only in the P2 high-dose group, but not in any of the P4-treated groups and, thus, only for the pristine graphene SLG sample, but not for the pristine graphene GNP sample. The other histopathological findings observed in the present study were interpreted to represent adaptive non-adverse findings.

## 4. Discussion

In the present study, we aimed to define a combined in vitro/in vivo approach to predict adverse lung effects of graphene-related nanoplates (GRNP), in particular, pristine single-layer graphene (SLG) and pristine graphene nanoplatelets (GNP), some of which have previously been shown to exhibit at least in vitro cytotoxic and genotoxic potential, depending, amongst others, on the specific material characteristics, the cell model used, the endpoints, concentrations and incubation times. For the prediction of adverse lung effects of graphene materials, it would, nevertheless, be highly desirable to have meaningful and differentiating in vitro screening approaches in place to enable prescreening of new materials before starting in vivo evaluations. 

Up to now, a variety of in vitro assays have been established to predict the in vivo lung toxicity of engineered nanoparticles in subacute intratracheal instillation or inhalation studies in rats. Often, commercially available permanent lung epithelial or alveolar macrophage cell lines are used as model systems. Cells are then exposed in a submerse manner to nanomaterial dispersions, or the test items are dosed as aerosols using air-liquid interface approaches. Acute lung inflammogenicity in a rat instillation model had previously been compared with certain in vitro toxicity endpoints (comprising cytotoxicity, pro-inflammatory cytokine expression or hemolytic potential) by [52]. Nanoparticles acting via soluble toxic ions (e.g., ZnO) showed positive results in most of the assays and were consistent with the lung inflammation data, whereas low soluble dust showed a good correlation only in the hemolysis assay. Wiemann et al. [53] selected the permanent rat alveolar macrophage cell line NR8383, which was also used in the present study, to investigate up to 18 inorganic nanoparticles. The authors were able to sort the test item panel into toxic and non-toxic subgroups and recommend only the biologically active nano-dusts for subsequent in vivo testing. Gorki et al. [54] described mouse ex vivo cultured alveolar macrophages (AM) as a suitable model, maintaining typical morphological features and expressing AM surface markers.

A combined in vitro/in vivo approach was reported by Sayes et al. [55], who compared three different cell culture systems, i.e., ((i) immortalized rat L2 lung epithelial cells; (ii) primary rat alveolar macrophages; (iii) co-cultures of (i) and (ii) regarding their capability to predict the cytotoxic potential and inflammogenicity of various samples of dust in vivo (here using intratracheal instillation in the rat model). The authors only found an unsatisfactory correlation. However, they were still convinced that the approach could be successful in principle but concluded that there was a need for better culture systems. 

In the PLATOX project presented here, the aim was to establish an unpretentious yet robust and well reproducible in vitro cell model able to deliver a good correlation of results as compared to short-term inhalation tests in rats. Additional considerations were to focus on a stable, easily accessible cell system, with preferably primary, non-transformed cells. In this context, AM, representing primary cells of the lung, were chosen and harvested from non-treated rats. These cells were finally shown to exhibit better predictivity than the immortalized macrophage models. The combination of in vitro screening assays included both cytotoxicity (membrane damage, cell proliferation and metabolic activity) and DNA damage (DNA strand break induction) testing, thus covering decisive entry-level tests to allow the commercialization of new materials. Notably, in contrast to cytotoxicity screening, both AM and NR8383 cells were able to predict induction of DNA strand breaks in BAL cells, observed in vivo in the 28-day inhalation study on day 29 post-exposure. However, due to the monoculture type of the macrophage models, the in vitro models were not able to predict the trend toward oxidative DNA damage, most likely representing secondary genotoxicity due to ongoing inflammation. Irrespective of the chemical nature of pristine graphene, which is rather inert in principle, genotoxicity is nevertheless a topic to be considered. However, the exact mechanisms, besides secondary genotoxicity based on inflammation and production of reactive oxygen species, still have to be determined [14,56]. Notably, in a study by Ursini et al., some genotoxicity was detected in workers with occupational exposure to graphene by using the FPG-modified comet assay and analysis of micronucleus frequencies [57]. 

The induction of micronuclei by pristine graphene nanoplates and, thus, of fixed DNA damage was furthermore demonstrated in THP-1 cells, underlining the need to include genotoxicity testing in the screening of pristine graphene nanoplates [45]. The same robust endpoints could be mirrored and validated in the in vivo inhalation testing using cytotoxicity, differential cell count in lung lavage fluid as well as histopathological equivalents. 

In the DRF study with intratracheal instillation, the single-layer graphene P2 induced a statistically significant increase in eosinophils at day 3 post-treatment, whereas carbon black did not (Figure 3). Interestingly, this effect was not observed in the subsequent 28-day inhalation study. An increase in eosinophils in BAL fluid is not regularly found in powder studies with rats; however, a paper by Lee et al. [58] reported similar effects after intratracheal instillation of nickel oxide nanoparticles. Inflammatory cells were evaluated at days 1, 2, 3 and 4 post-treatment. The authors concluded that NiO nanoparticles in rats induced a unique mixed type of neutrophilic and eosinophilic inflammation 3 and 4 days after instillation, which was consistent with the inflammation by NiCl_2_ at day 1 after instillation. The mechanism of eosinophilia recruitment by nano-NiO seemed to be based on the direct rupture of cells, releasing a significant level of intracellular eotaxin. Notably, GRNP, in particular those with a low layer number, are thought to exhibit sharp edges, potentially enabling membrane damage. Perhaps a bolus application by intratracheal instillation might have triggered a NiO-comparable effect for the P2 SLG sample. A strong, concentration-dependent membrane-damaging effect was also observed for P2 in the AM in vitro model.

Moghimian and Nazarpour [59] reported an acute inhalation test performed under Good Laboratory Practice (GLP) at Charles River Laboratories Montreal using graphene powder with 6–10 graphene layers. The maximally feasible aerosol concentration of 1900 mg/m^3^ was found as the NOAEL value, thus showing the same low in vivo toxicity as found for the graphene nanoplatelet sample P4 in the present study. Kim et al. [21] investigated a commercial multilayer graphene (Cabot Corp, USA; GPX-205; 20–30 layers) in a 28-day inhalation test at aerosol concentrations of 0.12, 0.47 and 1.88 mg/m^3^. No dose-dependent effects were recorded for body weights, organ weights, BAL fluid inflammatory markers and blood biochemical parameters at days 1 and 28 post-exposure. No distinct lung pathology was observed at days 1, 28 and 90 post-exposure, suggesting low toxicity and a NOAEL of no less than 1.88 mg/m^3^. These results are also in agreement with the outcome of P4 in the 28-day inhalation study in the present PLATOX project that used a similar dosing scheme.

In another study, mice were exposed to graphene nanoplates by pharyngeal aspiration at doses of 4–40 µg/mouse [60]. Three types with lateral sizes of 1, 5 or 20 µm were analyzed. At the high dose, increased lung inflammation was induced in lavage fluid to a higher degree by the 5- and 20-µm-sized platelets (G5, G20) than observed for the 1-µm-sized (G1) and the carbon black reference. G5 and G20 showed no or minimal lung epithelial hypertrophy and hyperplasia and no development of fibrosis at 2 months post-exposure. Interestingly, with regard to fibrosis, the MRC-5 lung fibroblasts, used as one in vitro screening model in the present study, were shown to lack significant uptake of GRNP and induction of membrane damage.

For the new material class of GRNP, appropriate risk characterization is presently missing due to the limited database. In the PLATOX project, the highest biological response both in vitro and in vivo was detected for the single-layer graphene. All other GRNP showed a weaker or irrelevant toxic impact. According to literature searches, the modified GRNP (e.g., CG, GO) of the in vitro test item set are relatively well toxicologically characterized, with, e.g., CG exhibiting, in contrast to pristine graphene platelets, a higher hydrophilicity, supposed to result in a lower disturbance of cells and subsequent cytotoxicity [44], and to enable better clearance. Therefore, it was decided to use another non-functionalized, i.e., the multi-layer graphene P4 (GNP), as a counterpart for the final validation test in vivo. 

Thus, the main output of this study is a justified step forward toward a validated in vitro (geno)toxicity screening tool for unmodified/non-functionalized graphene nanoplate species based on AM as a cell model. The screening tool, however, should be further supplemented by endpoints that more concretely mirror the liberation of pro-inflammatory mediators, with eicosanoids, TXB2 in particular, representing a promising candidate. Additionally, with regard to the dispersion of nanomaterials in protein-containing media for in vitro screening, protein corona should be considered to potentially have an impact on screening outcomes. Any pristine particle interacting with a biological medium forms a protein corona on its surface. However, its potential influence on particle toxicity is still strongly under debate, as the protein corona is not equivalent to a covalent technical coating that could hide particle surface-mediated toxicity excessively in the respiratory tract or in cell line assays [61].

Finally, the screening approach used predicted conclusively the adverse findings observed in the in vivo inhalation test. Based on these findings, the following toxicological ranking of single-layer (SLG) vs. multilayer graphene (GNP) was derived, as compared to the carbon black reference (Printex 90): SLG > MLG > P90. Based on BAL fluid analysis (PMN percentage) and histopathological examination (granulocytic infiltration), a NOAEC of 0.8 mg/m^3^ was finally derived for the investigated SLG. The evaluated GNP sample and CB reference, however, were weaker in the toxic response and the calculated NOAEC amounted to >3.2 mg/m^3^.

## 5. Conclusions

The present study has shown that the graphene nanoplatelet sample P4 (GNP; multilayer graphene) had almost no lung toxicity/pro-inflammatory potential following a 28-day inhalation study, in contrast to a moderate effect found with the single-layer graphene nanoplates P2 (SLG). When considering these in vivo results with a clear cytotoxic, genotoxic and pro-inflammatory potential of SLG, but less activity of GNP after 28 days of inhalation, alveolar macrophages, as primary rat cells derived from the respiratory tract, showed the highest reliability in predicting the in vivo adverse lung effects of the tested pristine graphene nanoplates, as members of the graphene-related two-dimensional (2D) nanomaterials group (GRNP). This includes lactate dehydrogenase (LDH) release and the induction of DNA strand breaks as the most meaningful endpoints. It can be concluded from the study results that, in principle, a predictive in vitro lung-focused toxicity screening of GRNP seems possible. However, relevant cell types and endpoints, as well as appropriate culture conditions, incubation times, and concentrations, should be chosen. Furthermore, the photometric and immunological detection methods should always be carefully adapted to GRNP properties. 

## Figures and Tables

**Table 1:** GRNP materials, codes, providers, identification, characteristics and synthesis.

Code	Material	Supplier/ Identification	Diameter [µm] *	Thickness [nm] *	BET Surface Area [m^2^/mg]	Dispersible in *	Preparation/Properties *
P1	Single-Layer Graphene (SLG)	ACS: GN1P0005	0.4–5	0.6–1.2	278 (400–1000) *	Water, ethanol and others	Thermal exfoliation + hydrogen reduction; sulfur impurities
P2	Single-Layer Graphene, (Graphene factory) (SLG)	ACS: GNP1F010	0.5–5	1–5 atomic layers	620 (650–750) *	No information	Thermal exfoliation + hydrogen reduction
P3	Carboxyl Graphene (CG)	ACS: GNCP0005	1–5	0.8–1.2	1.5	Polar solvents like DMF	(1) Modified Hummer’s Method to make graphene oxide (2) Conversion of (-OH) and (C-O-C) into (-COOH); non-conductivity
P4	Graphene Nanoplatelets (GNP)	ACS: GNNP0051	2–7	2–10	15 (20–40) *	Easy to disperse	Stacks of multilayer graphene; high aspect ratio (width to thickness); D50 = 48.93 µm, phosphorous impurities
P5	Graphene Oxide (S Method) (GO)	ACS: GNOS0010	1–15	0.8–1.2	5.2 (5–10) *	No information	Staudenmaier Method; oxygen content: 35 wt %; single-layer ratio > 90%
P6	Graphite Oxide	ACS: GTOP0002	0.5–5	1–3	2.7	Polar solvents like water, ethanol, DMF	Modified Hummer’s Method; oxygen content: 46 wt %; intercalated with either two ethanol or methanol monolayers
P7	Reference Graphene Nanoplatelets (GNP)	Avanzare: GR1	2	3	195 (70) *	No information	No XPS (low defects by Raman), all C1s; 8 ± 0.5 atomic graphene layers
P8	Spherical Carbon Black (CB)	Evonik: Printex 90^®^	14	n.a.	317 (337) *	No information	Spherical; specified as >99% pure carbon black, PAH = 0.039 ppm

* Values and information given by the supplier; BET = Brunauer–Emmett–Teller method; DMF = dimethylformamide; n.a. = not applicable.

**Table 2:** Dosing scheme of the 28-day nose-only inhalation test with 28 days of recovery.

	Clean Air	P2 Low	P2 Mid	P2 High	P4 Low	P4 Mid	P4 High	Printex 90^®^
Aerosol conc. [mg/m^3^]	0	0.2	0.8	3.2	0.2	0.8	3.2	3.2
MMAD [µm]	n.a.	1.92 B	2.52 B	3.11 M	-	2.72 B	2.63 B 3.87 B	0.92 M
GSD [–]	n.a.	(2.57)	(2.34)	(3.49)	-	(2.05)	(2.34) (3.12)	(3.53)

GSD: geometric standard deviation; n.a.: not applicable; M: Marple impactor; B: Berner impactor.

**Table 3:** GRNP-related histopathological findings at day 1 post-exposure.

Test Material Group				Clean Air	P2 Low	P2 Mid	P2 High	P4 Low	P4 Mid	P4 High	P8
Number of Animals				5	5	5	5	5	5	5	5
Lung	Accumulation of particle-laden macrophages	Alveolar	Occ.	0	5	5	5	2	4	5	5
			Grade	0	1	1.2	2	0.4	0.8	1.2	1.8
		Interstitial	Occ.	0	0	0	1	0	0	0	1
			Grade	0	0	0	0.2	0	0	0	0.2
		BALT	Occ.	0	0	0	2	0	0	0	4
			Grade	0	0	0	0.4	0	0	0	0.8
	Particle-laden giant cells (syncytia)		Occ.	0	0	0	5	0	0	1	0
			Grade	0	0	0	1	0	0	0.2	0
	Infiltration granulocytes	Alveolar	Occ.	0	0	0	4	0	0	0	0
			Grade	0	0	0	0.8	0	0	0	0
LALN	Accumulation of particle-laden macrophages		Occ.	0	0	0	0	0	0	0	3
			Grade	0	0	0	0	0	0	0	0.6
Nasal cavity	Eosinophilic globules	Olfactory epithelium	Occ.	0	1	1	3	0	0	1	0
			Grade	0	0.2	0.2	0.6	0	0	0.2	0

Occ. = Occurrence: number of animals showing the findings in the respective group; Grade: mean group grade of this lesion. Grades can range from 0 (normal) up to 5 (very severe change): 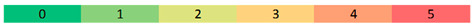.

**Table 4:** GRNP-related histopathological findings at day 29 post-exposure.

Test Material Group				Clean Air	P2 Low	P2 Mid	P2 High	P4 Low	P4 Mid	P4 High	P8
Number of Animals				5	5	5	5	5	5	5	5
Lung	Accumulation of particle-laden macrophages	Alveolar	Occ.	0	3	5	5	1	3	5	5
			Grade	0	0.6	1	1.6	0.2	0.6	1	1.4
		Interstitial	Occ.	0	0	0	3	0	0	0	1
			Grade	0	0	0	0.6	0	0	0	0.2
		BALT	Occ.	0	0	1	0	0	0	0	5
			Grade	0	0	0.2	0	0	0	0	1
	Particle-laden giant cells (syncytia)		Occ.	0	0	1	5	0	0	0	0
			Grade	0	0	0.2	1.6	0	0	0	0
	Infiltration granulocytes	Alveolar	Occ.	0	0	0	1	0	0	0	0
			Grade	0	0	0	0.2	0	0	0	0
LALN	Accumulation of particle-laden macrophages		Occ.	0	0	0	4	0	0	0	2
			Grade	0	0	0	0.8	0	0	0	0.4
Nasal cavity	Eosinophilic globules	Olfactory epithelium	Occ.	0	0	1	5	1	0	3	1
			Grade	0	0	0.2	1.4	0.2	0	0.6	0.2

Occ. = Occurrence: number of animals showing the findings in the respective group; Grade: mean group grade of this lesion. Grades can range from 0 (normal) up to 5 (very severe change): 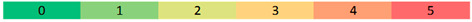.

## Data Availability

Not applicable.

## Supplementary Materials

The following supporting information can be downloaded at: https://www.mdpi.com/article/10.3390/nano12081254/s1, Figure S1. Time-dependent induction of membrane damage in primary rat alveolar macrophages by incubation with P2 (SLG); Figure S2. Uptake of GRNP by NR8383 and RAW 264.7 cells, as assessed by light microscopy or flow cytometry, respectively; Figure S3: TXB2 release from NR8383 cells, preliminary data; Figure S4: Differential cell counts in BAL fluid at day 1 and day 29 post-exposure of the 28-day nose-only inhalation study with the P2 (SLG), P4 (GNP) and P8 (CB) materials, in absolute cell numbers; Figure S5: Coefficient of variation (CV) of G0/G1 cell cycle peak of BALF cells from the 28-day nose-only inhalation study estimated by the FlowJo software. Table S1. Biochemical parameters analyzed in the BAL fluid of the DRF in vivo study.

## Abbreviations

AM	Primary rat alveolar macrophages
BAL	Bronchoalveolar lavage
β -Glu	β-Glucuronidase
BMD	Benchmark dose
BMD30	Benchmark dose 30%
BMR	Benchmark response
BMDL	Benchmark dose lower confidence limit
BMDU	Upper confidence limit of BMD
CB	Carbon black
CG	Carboxyl graphene
CNT	Carbon nanotubes
1D	One-dimensional
2D	Two-dimensional
3D	Three-dimensional
DAPI	4′,6-diamidino-2-phenylindole
DMEM	Dulbecco’s Modified Eagle Medium
DRF	Dose range finding
EMEM	Eagle’s Minimal Essential Medium
FCS	Fetal calf serum
FLG	Few-layer graphene
GFN	Family of graphene-based materials
GNP	Graphene nanoplatelets
GRNP	Graphene-related nanoplates
GO	Graphene oxide
GSD	Geometric standard deviation
HE	Hematoxylin and eosin
hOGG1	Human 8-oxoguanine DNA N-glycosylase 1
LALN	Lung-associated lymph nodes
LDH	Lactate dehydrogenase
LMA	Low melting point agarose
LPS	Lipopolysaccharide
MLG	Multilayer graphene
MMAD	Mass median aerodynamic diameter
MPPD	Multiple-Path Particle Dosimetry
MWCNT	Multiwalled carbon nanotubes
n.a.	Not applicable
NALT	Nasal-associated lymphoid tissue
NC	Negative control
NOAEL	No observed adverse effect level
8-OHdG	8-hydroxy-2′-deoxyguanosine
PI	Propidium iodide
PMN	Polymorphonuclear neutrophils
rGO	Reduced graphene oxide
RICC	Relative increase in cell count
SD	Standard deviation
SEM	Scanning electron microscopy
SLG	Single-layer graphene
TI	Tail intensity
TP	Total protein
TXA_2_	Thromboxane A_2_
TXB2	Thromboxane B_2_
