# Supplementary material for: PLATOX: Integrated In Vitro/In Vivo Approach for Screening of Adverse Lung Effects of Graphene-Related 2D Nanomaterials
Source: Nanomaterials (Basel). 2022 Apr 7;12(8):1254. doi: 10.3390/nano12081254 (PMC9028947; doi:10.3390/nano12081254)
Supplement: Supplementary file 1 [file nanomaterials-12-01254-s001.zip › nanomaterials-1639044-Supplementary Materials.pdf]

nanomaterials

Supplementary Materials:

PLATOX: Integrated in Vitro/in Vivo Approach for Screening of Adverse Lung Effects of Graphene-Related 2D Nanomaterials

Otto Creutzenberg [1,*], Helena Oliveira [2], Lucian Farcal [3], Dirk Schaudien [1], Ana Mendes [2], Ana Catarina Menezes [2], Tatjana Tischler [1], Sabina Burla [3,4] and Christina Ziemann [1,*]

1 Fraunhofer Institute for Toxicology and Experimental Medicine ITEM, 30625 Hannover, Germany; dirk.schaudien@item.fraunhofer.de (D.S.); tatjana.gripp@gmx.de (T.T.)
2 Department of Biology & CESAM, University of Aveiro, 3810-193 Aveiro, Portugal; holiveira@ua.pt (H.O.); anamendes@live.com.pt (A.M.); catarinamenezes@msn.com (A.C.M.)
3 BIOTOX SRL, 407280 Cluj-Napoca, Romania; lucian.farcal@biotox.ro (L.F.); sabina.burla@list.lu (S.B.)
4 Department of Environmental Research and Innovation, Luxembourg Institute of Science and Technology, 4422 Belvaux, Luxembourg
* Correspondence: otto.creutzenberg@item.fraunhofer.de (O.C.); christina.ziemann@item.fraunhofer.de (C.Z.); Tel.: +49-511-5350-461 (O.C.); +49-511-5350-203 (C.Z.)

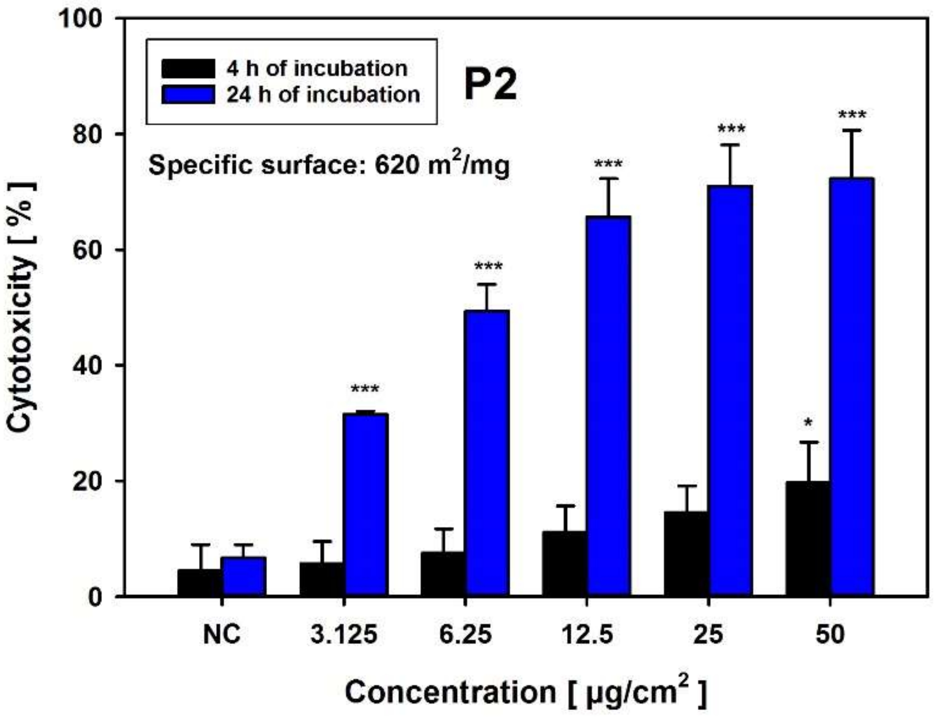

Figure S1. Time-dependent induction of membrane damage in primary rat alveolar macrophages by incubation with P2 (SLG). Cells were incubated without (NC) or with the given concentrations of P2 (SLG) for 4 or 24 h with subsequent measurement of LDH activity in the culture supernatants. Data represent arithmetic means ± SD of three independent experiments. */*** Statistically significantly different from NC: $p \leq 0.05$ or $p \leq 0.001$, respectively. Student's *t*-test for unpaired values, two-sided.

A

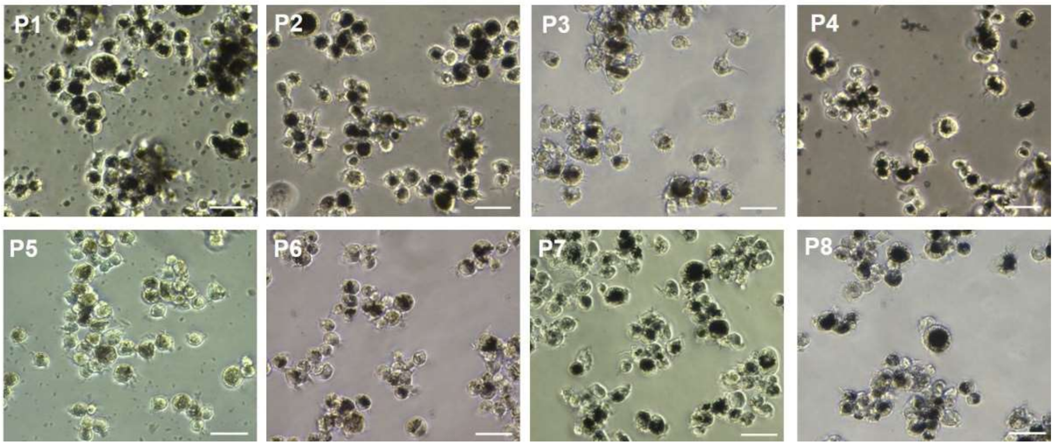

B

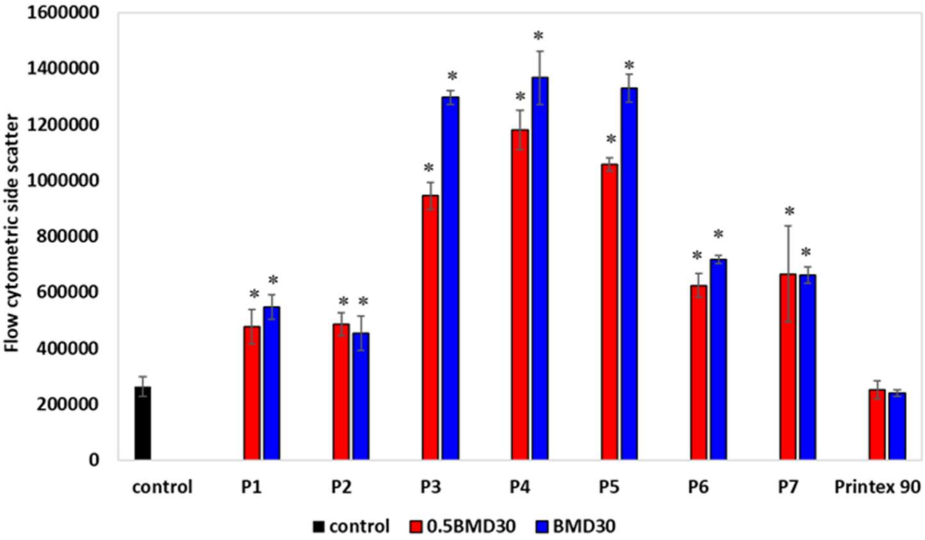

Figure S2. Uptake of GRNP by NR8383 and RAW 264.7 cells, as assessed by light microscopy or flow cytometry, respectively. (A) For assessment of GRNP uptake in NR8383 cells by light microscopy, cells were incubated for 24 h with 12.5 µg/cm^2 of the various GRNP dispersions. White bars in the pictures represent a distance of 50 µm. Cells are more or less filled with black GRNP material, depending on type of GRNP. (A) For assessment of GRNP uptake by flow cytometry RAW 264.7 cells were seeded (1 x 10^5 per well) in a 12-well plate and pre-incubated for 24 h at 37 °C in a humidified atmosphere with 5 % CO_2. The medium was then replaced by fresh medium (negative controls) or GRNP dispersions at the respective 0.5 x BMD30 and BMD30 concentrations. Cells were incubated for 24 h at 37 °C. After that, the supernatant was removed and cells were washed once with phosphate-buffered saline. Then, 1 mL of cell culture medium was added, cells were collected by scraping and analyzed flow cytometrically using an Attune® Acoustic Focusing Cytometer (ThermoFisher Scientific, Brunswick, Germany). The uptake of GRNP was finally measured by side-scatter signal (SS) analysis, which gives information on cell complexity. The uptake results were expressed as the mean ± SD versus control. * Indicates statistically significant differences between the cell culture negative control (control) at $p < 0.001$; Dunnett's test.

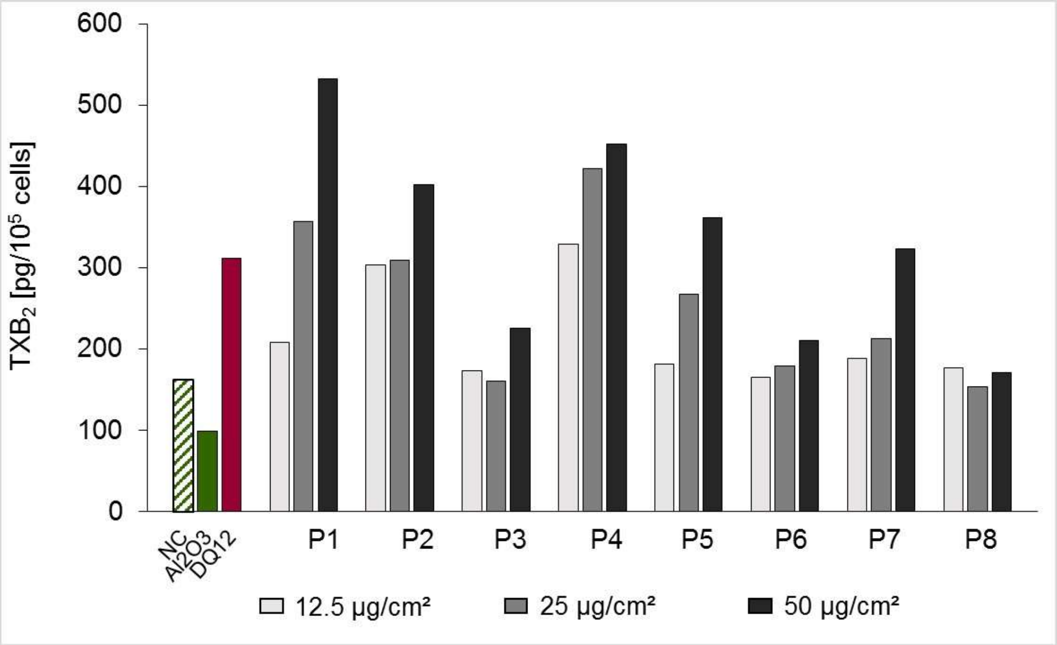

Figure S3. TXB2 release from NR8383 cells, preliminary data. NR8383 cells were seeded at a density of 24.5 x 10^4 cells per cm^2 in 24-well plates and incubated after 24 h of pre-culture without (NC, negative control) or with different GRNP concentrations (12.5, 25 and 50 µg/cm^2) for 24 h. Al_2O_3 served as particle-like negative and quartz DQ12 as particle-like positive control (both at 50 µg/cm^2). TXB_2 was then determined using a highly specific competitive ELISA kit (Cayman Chemical, Ann Arbor, USA), according to the manufacturers' instructions. Data represent arithmetic means of two independent experiments with duplicate determination each.

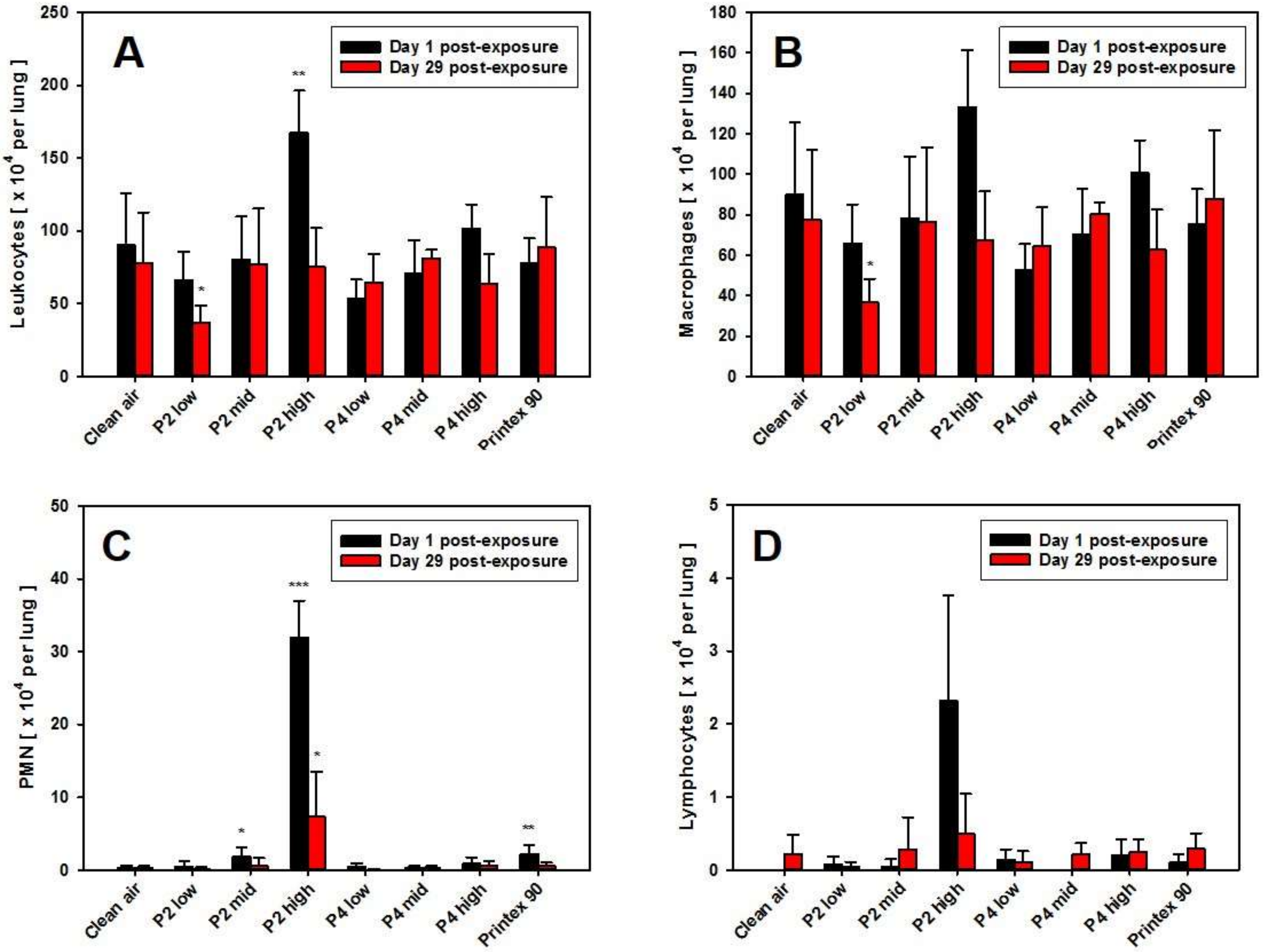

Figure S4. Differential cell counts in BAL fluid at day 1 and day 29 post-exposure of the 28-day nose-only inhalation study with the P2 (SLG), P4 (GNP) and P8 (CB) materials, in absolute cell numbers. (A) Total leukocytes; (B) Alveolar macrophages; (C) Polymorphonuclear neutrophils (PMN); (D) Lymphocytes. Data represent arithmetic means ± SD of 5 animals per treatment group. */**/*** Statistically significantly different from clean air negative control: $p \leq 0.05$, $p \leq 0.01$, or $p \leq 0.001$, respectively; Student's *t*-test for unpaired values, two-sided.

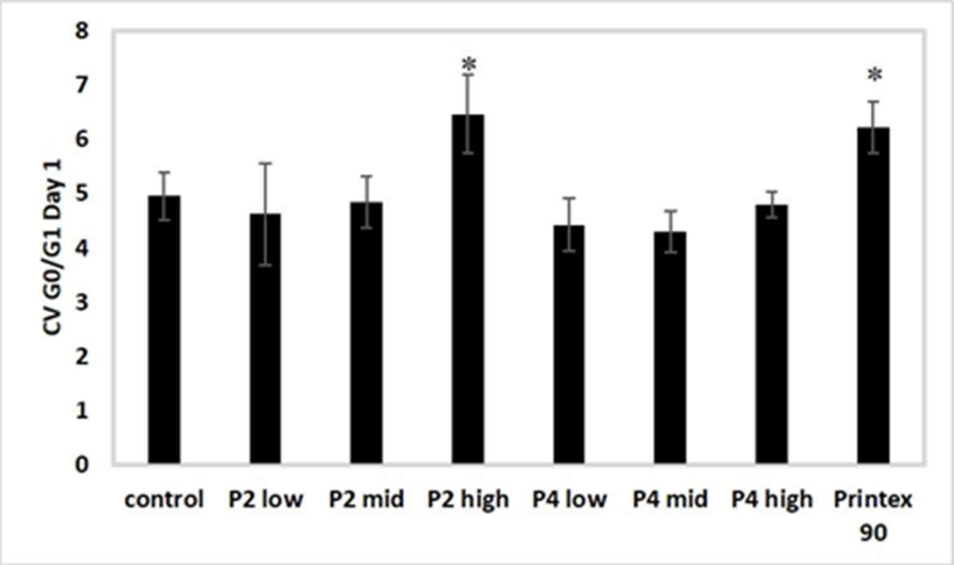

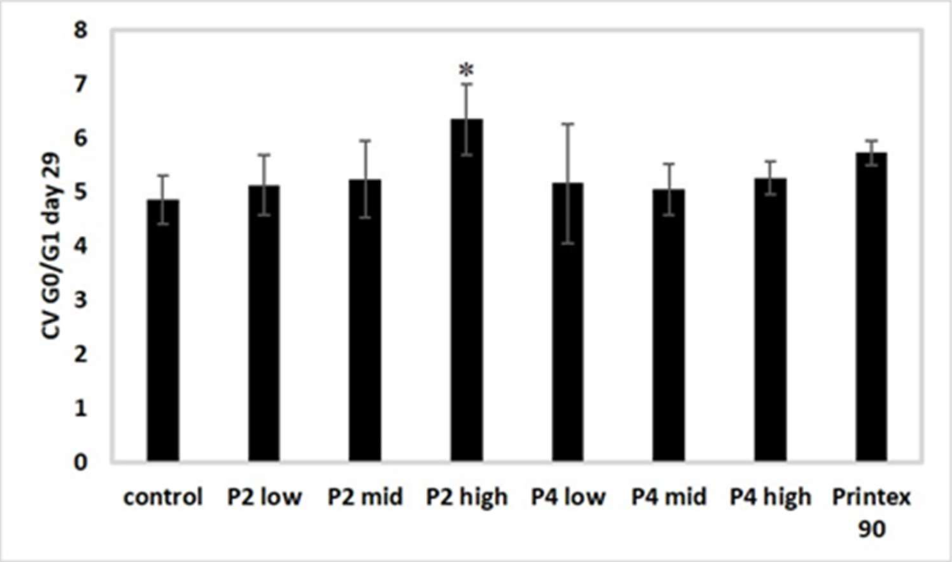

Figure S5. Coefficient of variation (CV) of G0/G1 cell cycle peak of BALF cells from the 28-day nose-only inhalation study at day 1 and day 29 post-exposure, estimated by the FlowJo software. Data represent arithmetic means ± SD of 5 animals per treatment group. * Statistically significantly different from clean air negative control: $p \leq 0.05$; Dunnett's test.

Table S1. Biochemical parameters analysed in the BAL fluid of the DRF *in vivo* study 3 days after intratracheal instillation of P2 (SLG), P4 (GNP) or P8 (CB).

**Treatment Dose (mg/rat)**		**LDH activity (U/L)**	**β-Glucuronidase (U/L)**	**Total protein (mg/L)**
Vehicle control (0.9% NaCl)	Mean	70.6	0.46	138.6
	SD	17.1	0.09	47.1
P2-SLG low 0.02	Mean	86.4	0.42	222.2
	SD	20.4	0.13	18.9
P2-SLG high 0.2	Mean	198.6**	0.98**	478.2**
	SD	72.5	0.37	183.8
P4-GNP low 0.02	Mean	76.4	0.38	190.2
	SD	9.2	0.11	37.0
P4-GNP high 0.2	Mean	160.4*	0.68	357.8*
	SD	93.6	0.18	190.6
P8-Carbon black (Printex® 90) 0.2	Mean	89.4	0.52	163.6
	SD	17.2	0.08	24.4

N = 5 animals per treatment group. */**/*** Statistically significantly different from vehicle control:
$p \leq 0.05$ or $p \leq 0.01$, respectively; Dunnett's test.
